# The BET PROTAC inhibitor dBET6 protects against retinal degeneration and inhibits the cGAS-STING in response to light damage

**DOI:** 10.1186/s12974-023-02804-y

**Published:** 2023-05-22

**Authors:** Xingfei Zhu, Wei Liu, Xiangcheng Tang, Yulin Chen, Xiangyu Ge, Qin Ke, Xingmiao Liang, Yuwen Gan, Yingfeng Zheng, Ming Zou, Mi Deng, Yizhi Liu, David Wan-Cheng Li, Lili Gong

**Affiliations:** 1 State Key Laboratory of Ophthalmology, Zhongshan Ophthalmic Center, Sun Yat-Sen University, Guangzhou, 510060 Guangdong China; 2 Shenzhen Key Laboratory of Ophthalmology, Shenzhen Eye Hospital, Jinan University, Shenzhen, China; 3 Peking University International Cancer Institute, Health Science Center, Peking University, Beijing, China; 4 Peking University Cancer Hospital and Institute, Peking University, Beijing, China

**Keywords:** Retinal degeneration, BET, Neuroinflammation, PROTAC, cGAS-STING, Microglia/macrophage

## Abstract

**Background:**

Chronic inflammation significantly contributes to photoreceptor death in blinding retinal diseases such as age-related macular degeneration (AMD) and retinitis pigmentosa (RP). Bromodomain and extraterminal domain (BET) proteins are epigenetic readers that act as key proinflammatory factors. We recently found the first-generation BET inhibitor JQ1 alleviated sodium iodate-induced retinal degeneration by suppressing cGAS-STING innate immunity. Here, we investigated the effects and mechanism of dBET6, a proteolysis‑targeting chimera (PROTAC) small molecule that selectively degrades BET by the ubiquitin‒proteasome system, in light-induced retinal degeneration.

**Methods:**

Mice were exposed to bright light to induce retinal degeneration, and the activation of cGAS-STING was determined by RNA-sequencing and molecular biology. Retinal function, morphology, photoreceptor viability and retinal inflammation were examined in the presence and absence of dBET6 treatment.

**Results:**

Intraperitoneal injection of dBET6 led to the rapid degradation of BET protein in the retina without detectable toxicity. dBET6 improved retinal responsiveness and visual acuity after light damage (LD). dBET6 also repressed LD-induced retinal macrophages/microglia activation, Müller cell gliosis, photoreceptor death and retinal degeneration. Analysis of single-cell RNA-sequencing results revealed cGAS-STING components were expressed in retinal microglia. LD led to dramatic activation of the cGAS-STING pathway, whereas dBET6 suppressed LD-induced STING expression in reactive macrophages/microglia and the related inflammatory response.

**Conclusions:**

This study indicates targeted degradation of BET by dBET6 exerts neuroprotective effects by inhibiting cGAS-STING in reactive retinal macrophages/microglia, and is expected to become a new strategy for treatment of retinal degeneration.

**Supplementary Information:**

The online version contains supplementary material available at 10.1186/s12974-023-02804-y.

## Introduction

The degeneration of photoreceptors is a shared feature of several major blinding retinal diseases, such as atrophic age-related macular degeneration (AMD) and retinitis pigmentosa (RP) [1, 2]. Inflammation is a key contributor to photoreceptor degeneration, and the accumulation and activation of macrophages/microglia have been detected in the retinal lesions of AMD and RP patients [3, 4]. In these retinal degenerative diseases, reactive macrophages/microglia have been found to actively phagocytose live photoreceptors and secrete proinflammatory cytokines and chemokines, which can accelerate photoreceptor death and visual loss [3, 4]. Therefore, modulating retinal inflammation and macrophage/microglial activity has been shown to be a promising therapeutic strategy for AMD and RP treatment [5–7].

Bromodomain and extraterminal (BET) family proteins are transcription transactivators and key coactivators of inflammatory gene transcription [8, 9]. These proteins contain four family members: BRD2, 3, 4 and BRDT; BRD2, 3, 4 are abundantly and ubiquitously expressed, while BRDT is testis-specific [10]. Inhibition of BET proteins by BET competitive inhibitors mitigates several inflammatory diseases, including retinal inflammation in *Pde6b*^rd10^ mutation [11, 12]. We recently found BRD4 was induced in mouse retina and human retinal pigment epithelium (RPE) cells upon oxidative stress, while the BRD4 competitive inhibitor JQ1 prevents retina and RPE degeneration, inhibits retinal inflammation and immune cell accumulation in retina and subretinal spaces [13].

JQ1 occupies the acetyl lysine-binding pocket of BET to release BET proteins from chromatin, resulting in the inhibition of target gene transcription [14]. However, increased BRD4 protein levels were observed in response to JQ1 treatment, which may compromise the effect of JQ1 [15]. Recently, BET PROTACs (proteolysis targeting chimera) that promote ubiquitination and proteasomal degradation of BET proteins, allowing prolonged and profound depletion of BETs, are presented as emerging technique for therapeutic intervention [16, 17]. Here, we selected dBET6, which is a bifunctional small molecule composed of a phthalimide moiety, a ligand for the E3 ubiquitin ligase cereblon, linked to JQ1 to target BET [18]. Compared with the first-generation PROTAC dBET1, dBET6 shows tenfold more potency in degrading BRD4 and improved cell permeability [18], although whether it can cross the blood‒retina barrier and degrade retinal BET is unknown.

Through cytosolic DNA sensing, the cyclic GMP-AMP synthase-stimulator of interferon genes (cGAS-STING) pathway acts as a key mediator of inflammation in response to infection, cellular stress and tissue damage [19–21]. After binding to cytosolic DNA, cGAS catalyzes the synthesis of the second messenger cyclic dinucleotide 2′-3′-cGAMP (cGAMP), which activates STING, and induces production of type I interferons via IRF3 or IRF7 and other inflammatory cytokines via the NFκB pathway [22, 23]. Aberrant activation of STING is involved in multiple autoimmune diseases [24]. Recently, the pathogenic effects of cGAS-STING in neuroinflammation have emerged. In brain microglia, activation of cGAS-STING signaling has been shown to contribute to neuronal degeneration [25, 26]. Suppression of STING in microglia is neuroprotective in tau-induced brain inflammation or α-synucleinopathy [25, 26].

We and others have recently shown that cGAS-STING signaling is aberrantly activated in retina and RPE of AMD patients, and contributes to RPE and retinal degenerations [13, 27, 28]. We further showed that JQ1 epigenetically silenced *STING* transcription and thus reduced retinal inflammation in sodium iodate (SI) injury [13]. However, which type of retinal cells express cGAS-STING, and whether BET inhibitors regulate cGAS-STING in cell-type specific manner are unknown.

Here, we used a light-induced retinal degeneration model. Light damage (LD) is an environmental risk factor for the degeneration of photoreceptors, which are unique light-sensitive neurons. Intraperitoneal (I.P.) injection of dBET6 rapidly degraded retinal BET proteins. dBET6 preserved retinal structure and function and inhibited retinal inflammation in response to LD. Mechanistically, the RNA-sequencing (RNA-seq) results showed the dramatic induction of cGAS-STING pathway after LD. Furthermore, analysis of published single-cell (sc) RNA-seq results [29] revealed that cGAS and STING are mainly expressed in retinal microglia, and we found administration of dBET6 reduced STING expression in reactive retinal macrophages/microglia after LD. To our knowledge, this is the first evidence showing BET PROTAC-mediated protection in a retinal degenerative disease model, which should provide new therapeutic strategies for treating AMD and RP.

## Methods

### Experimental animals

BALB/cJ and C57BL/6J mice (5–8 weeks) were purchased from Sun Yat-Sen University Laboratory Animal Center. Mice were housed under a 12:12 light:dark cycle with ad libitum access to standard mouse chow and both female and male mice were randomly used in the experiments. When eye was referred, it denotes the eye of each individual mouse.

### Light exposure

The mice were placed in constant darkness 12 h before exposure to light. Nonanesthetized mice were then exposed to typical laboratory lighting (50 lx) or white light using light emitting diode (LED) lights placed at top a standard mouse cage. The light intensities used were 15,000 lx for BALB/cJ mice and 30,000 lx for C57BL/6J mice, both with an exposure time of 2 h. An average of 2–3 mice per cage was used for each experiment. Prior to light exposure, C57BL/6J mice were treated with 1–2 drops of Tropicamide Phenylephrine Eye Drops to dilate their pupils.

### Intraperitoneal drug administration

dBET6 was prepared in 5% DMSO, 30% Polyethylene glycol 300 and 5% Tween 80 at concentrations of 1 μg/μl. Buffer used for dBET6 preparation served as vehicle control. dBET6 or vehicle control administered to mice 1 h prior to light exposure and 24 h after light exposure by intraperitoneal injection (i.p). To determine the potential effects of vehicle injection on retinal morphology and function, BALB/cJ mice were i.p. injected with vehicle or left untreated (CTRL). After 48 h, optical coherence tomography (OCT), electroretinography (ERG) and hematoxylin and eosin (HE) staining were performed on CTRL and vehicle mice.

### RNA-seq analysis

Mice were treated as described in Fig. 2A. The RNA isolation was conducted according to the manufactory’s protocol. Briefly, two eyes from the same mouse were collected and directly lysed in 1 mL of TRIzol™ reagent. The retinas were homogenized by vortexing for 30 s and 0.2 mL of chloroform was added. After centrifugation, the aqueous phage was collected and 0.5 mL of isopropanol was added. The RNA was precipitated in − 20 °C overnight with addition of 5 μg glycogen. After centrifugation, the RNA was washed by 75% ethanol and dissolved in RNase-free water. The purified RNAs were then sent to Chi Biotech company for sequencing. Bulk RNA-seq data can be accessed from NCBI Sequence Read Archive (SRA) database under accession number PRJNA916821 and PRJNA954826. Aadapters were removed by Trim Galore v1.18. Raw sequencing data were mapped to the GRCm39 genome assembly using HISAT2 v2.2.1 [22]. We calculated fragments per kilobase of exon per million (FPKM) by featureCounts v2.0.1 [23]. Using DESeq2 [24], we identified differentially expressed genes (DEGs) between control and light damage groups (fold change ≥ 2 and FDR ≤ 0.05). R package “clusterProfiler” was used for function enrichment analysis on genes.

### Retinal flat mount

The mouse eyeball was rinsed in PBS and briefly fixed in 4% Paraformaldehyde (PFA) for 5 min. After washing with PBS, the cornea and lens were removed, and the retina was carefully separated from retinal pigment epithelium/choroid membrane. The retinas were then fixed in 4% PFA for additional 15 min at room temperature (RT). After washing with PBS, the retinas were permeabilized by 0.3% PBST (PBS + 0.3%triton x-100) for 15 min, blocked with 2.5% BSA (prepared in PBST) for 30 min, RT. The retinas were then proceeded to standard incubation of the primary and secondary antibodies, which were prepared in blocking buffer. Finally, the retinas were flattened onto a slide, and mounted with anti-fade mounting medium. Images were acquired using the a LSM980 confocal microscope (Carl Zeiss, German). The process length and endpoints of microglia/macrophages were measured by using Image J according to previous report [30].

### scRNA-seq data analysis

The scRNA-seq data were available at GitHub (https://github.com/jiewwwang/Single-cell-retinal-regeneration). We removed low-quality cells (< 200 genes, > 6000 genes or mitochondrial genes > 10%). R package Seurat was used to perform clustering analysis of single cells. Clusters were visualized using t-distributed stochastic neighbor embedding (tSNE). Each cluster of cells was annotated by known marker genes from cellmarker2.0 (http://bio-bigdata.hrbmu.edu.cn/CellMarker/index.html) [31]. Microglia cells were selected by the special markers (Csf1r, Cx3cr1, Hexb, C1qa, P2ry12, Aif1, Trem2 and Tmem119 for mouse, C1QA, TMEM119, CD163 and AIF1 for human). All known marker genes were reported in mouse or human.

### High-content analysis

Cell motility was analyzed using the High Content Analysis System (ImageXpress Micro 4) as previously described [13]. BV2 cells were seeded at 1 × 10^5^ cells/well in 12-well plate and treated with indicated drug. For cell motility assay, live cells were recorded every 5 min for 8 h by with the channel of Digital Phase Contrast with 20× objective lens.

### Automated western immunoblotting (WES) analysis

WES is a fully automated protein expression analysis system and is especial for micro-content protein. The retinas were dissected in PBS and suspended in 150 μl of radioimmunoprecipitation (RIPA) assay buffer (per retina) containing proteinase inhibitor cocktail and protein phosphatase inhibitor as described before [13]. For WES analysis, a final concentration of 1 μg/μl of protein sample was used. The detailed procedure was performed according to the manufacturer’s instructions (Protein Simple). The uncropped images are shown in Additional file 2: Fig. S2.

### Immunofluorescence (IF) staining and in situ terminal deoxynucleotidyl transferase dUTP nick end labeling (TUNEL) assay

After euthanasia, eyeballs were enucleated into PBS to remove excess connective tissue and fixed in 4% PFA for 10 min, a small cut was made in the cornea of each mouse eye after fixation for 10 min and further fixation for 50 min. The eye was then dehydrated using 10% and 20% sucrose in 0.1 M phosphate-buffered saline for 1 h and dehydration in 30% sucrose overnight, and then eyes in 30% sucrose and optimal cutting temperature (OCT) 1:1 mixture for 1 h before embedded in OCT medium. Retinal cryosections along the superior and inferior retinal meridians were cut at 8-μm thickness and then proceed to standard immunofluorescence protocols as previously described [13]. The antibodies are listed in Table 1. TUNEL assay was performed according to the manufactory’s procedure. Stained retinal sections were imaged with TissueFAXS microscopy.

**Table 1:** Key reagents and resources used in this study

Reagent or resource	Source	Identifier	Dilutions
*Antibodies*			
BRD2 polyclonal antibody	Proteintech	cat#22236-1-AP	Wes 1:40
BRD3 polyclonal antibody	Proteintech	cat#11859-1-AP	Wes 1:40
Rabbit polyclonal to Brd4	Abcam	cat# ab84776	Wes 1:40;WB 1:1000
Rabbit monoclonal [EPR16588] to IBA1	Abcam	cat#ab178846	WB 1:1000;IF 1:200
Beta tubulin mouse monoclonal antibody	Proteintech	cat#66240-1-Ig	Wes 1:10,000;WB 1:5000
Anti-rabbit IgG (H + L), F(ab′)2 fragment (Alexa Fluor® 488 conjugate)	Cell Signaling Technology	cat# 4412	IF 1:500
Anti-mouse IgG (H + L), F(ab′)2 fragment (Alexa Fluor® 594 conjugate)	Cell Signaling Technology	cat# 8890	IF 1:500
Anti-rabbit IgG (H + L), F(ab′)2 fragment (Alexa Fluor® 594 conjugate)	Cell Signaling Technology	cat# 8889	IF 1:500
Rabbit polyclonal to GFAP	Affinity Bio sciences	cat# DF6040	WB 1:1000
GFAP polyclonal antibody	Proteintech	Cat No. 16825-1-AP	IF 1:200
cGAS (D3O8O) rabbit mAb (mouse specific)	Cell Signaling Technology	cat#31659	WB 1:1000
TBK1/NAK (D1B4) rabbit mAb	Cell Signaling Technology	cat#3504	WB 1:1000
Phospho-TBK1/NAK (Ser172) (D52C2) XP® rabbit mAb	Cell Signaling Technology	cat#5483	WB 1:1000
*p*-Histone H2A.X (Ser 139) anti-mouse	Santa Cruz Biotechnology	cat#sc-517348	WB 1:1000
STING (D1V5L) rabbit mAb (rodent preferred)	Cell Signaling Technology	cat#50494	WB 1:1000;IF 1:200
CoraLite®594-conjugated TMEM173/STING polyclonal antibody	Proteintech	CL594-19851-100UL	IF 1:200 for retinal flat mount
TMEM173/STING monoclonal antibody	Proteintech	cat#66680-1-Ig	IF 1:200
Rabbit polyclonal to CD86	Affinity Bio sciences	cat# DF6332	WB 1:1000;IF 1:200
dsDNA Marker (HYB331-01)	Santa Cruz Biotechnology	cat#sc-58749	IF 1:200
CoraLite®594-conjugated TMEM173/STING polyclonal antibody	Proteintech	cat#CL594-19851	IF 1:200
CoraLite® Plus 488-conjugated IBA1 polyclonal antibody	Proteintech	cat#CL488-10904	IF 1:200
APC anti-mouse CD86 (GL1)	Proteintech	cat#APC-65068	IF 1:200
*Chemicals, peptides, and recombinant proteins*			
PEG300	Selleck	cat#S6704	
Tween-80	BioFroxx	cat#1716	
DMSO	MP Biomedicals	cat#196055	
LPS	Sigma	cat#L4391	
TUNEL BrightRed apoptosis detection kit	Vazyme	cat#A113	
dBET6	Selleck	cat#S8762	
MG132	Selleck	cat#S2619	
*Experimental models: cell lines*			
661W	Huangxuan Shen Lab	N/A	
BV2	ATCC		
*Experimental models: organisms/strains*			
Mouse: C57BL/6: C57BL/6J	Sun Yat-Sen University	C57BL/6J	
	Laboratory Animal Center		
Mouse: BALB/c	Sun Yat-Sen University	BALB/cJ	
	Laboratory Animal Center		
*Published sequencing data*			
Single-cell RNA-seq Data	GitHub	https://github.com/jiewwwang/Single-cell-retinal-regeneration	
Bulk-RNA Data	NCBI	PRJNA916821;PRJNA954826	
*Software and algorithms*			
GraphPad Prism 8.0.2	GraphPad software	https://www.graphpad.com/	
ImageJ 1.46/Fiji	NIH	https://imagej.nih.gov/ij/	
SRA Tools	NCBI	https://github.com/ncbi/sra-tools	
SAMTools	Li H et al. (2009)	http://www.htslib.org/	
featureCounts	Yang Liao et al. (2013)	http://www.bioconductor.org	
DESeq2	Michael I Love et al. (2014)	http://www.bioconductor.org/packages/release/bioc/html/DESeq2.html	
ComplexHeatmap	Zuguang Gu et al. (2016)	http://www.bioconductor.org/packages/devel/bioc/html/ComplexHeatmap.html	
clusterProfiler	Guangchuang Yu et al. (2012)	http://bioconductor.org/packages/release/bioc/html/clusterProfiler.html	
Seurat	Hao*, Hao*, et al. Cell (2021)	Tools for Single Cell Genomics **·** Seurat (satijalab.org)	

### Mouse retina protein extraction and Western blot (WB) analysis

The retinas were dissected in PBS and suspended in 150 μl of RIPA (per retina) containing proteinase inhibitor cocktail and protein phosphatase inhibitor as described before [26]. The total proteins were extracted sonication using an EpiSonic 2000 Sonication System (EPIGENTEK, Farmingdale, NY, USA) (Amplitude: 40%, 5 s on and 5 s off for 5 min in total). For WB analysis, 25–50 μg of total protein was used. The uncropped WB images are shown in Additional file 2: Fig. S2.

### Hematoxylin and eosin (HE) staining of retina

After euthanasia, the eyeballs were enucleated and fixed in FAS eye fixation solution (Servicebio#G1109) for 24 h. After fixation, the eye was then dehydrated in 60%, 70%, 80%, 90%, 100% ethanol and processed for paraffin embedding. Paraffin sections 8 μm thick were subjected to hematoxylin and eosin (HE) staining as previously described [13]. Stained retinal sections were imaged with TissueFAXS microscopy.

### Quantitative reverse transcription-PCR (qRT-PCR)

Both RT-PCR and qPCR were conducted according to the manufactory’s procedure. Total RNA was extracted wherein the genomic DNA was removed by DNase I digestion. For cDNA synthesis, 1 μg of total RNA was used. The gene expression levels were analyzed using ChamQ SYBR Color qPCR Master Mix (Vazyme #Q411-02) and the LightCycler® 480 System (Roche). The assays were performed in triplicate and the Ct values were normalized to that of beta actin or GAPDH as indicated. The primers used are listed in Table 2.

**Table 2:** Primers used for qRT-PCR

Gene and species	Primer direction	Primer sequence
Mouse IL1β	F	TGCCACCTTTTGACAGTGATG
Mouse IL1β	R	AAGGTCCACGGGAAAGACAC
Mouse iNOS	F	CCCTTCAATGGTTGGTACATGG
Mouse iNOS	R	ACATTGATCTCCGTGACAGCC
Mouse IL6	F	GTTCTCTGGGAAATCGTGG
Mouse IL6	R	CTGCAAGTGCATCATCGTT
Mouse CCL2	F	GGTGTCCCAAAGAAGCTGTAGT
Mouse CCL2	R	TTCCGATCCAGGTTTTTAATGT
Mouse TNF	F	ACGGCATGGATCTCAAAGAC
Mouse TNF	R	AGATAGCAAATCGGCTGACG
Mouse GAPDH	F	CCACTTGTGGCAAGAGGCTA
Mouse GAPDH	R	GTGGAGAGTTGGGACGTGAG

### Cell culture and treatment

The BV2 cells and 661W cells used in this study were authenticated by STR profiling and have been tested for mycoplasma contamination. Cells were cultured in DMEM containing 10% fetal bovine serum and 1% penicillin–streptomycin. Lipopolysaccharide (LPS) (10 ng/ml) and mouse IFN-γ (20 ng/ml) was used to activate immune cells. For MG132 treatment, 661W cells were pretreated with MG132 (50 μM) for 2 h and then dBET6 (100 nM) was added for additional 2 h before collection for WB analysis.

### Optical coherence tomography (OCT)

Before imaging, mice were anesthetized by 1% pentobarbital sodium (70 μl/10 g, prepared in normal saline solution), pupils were dilated with 1–2 drops of Tropicamide Phenylephrine Eye Drops and the cornea was lubricated with normal saline solution. OCT was performed on both eyes with a Heidelberg, Spectralis OCT device (Heidelberg Engineering) to investigate structural changes. Thickness measurements were performed with a circular ring scan (circle diameter 1, 3, 6 ETDRS), centered on the optic nerve head, which represents the average retinal thickness in a certain field. Central retinal thickness was calculated from four fields around the optic nerve head using the Heidelberg Eye Explorer Software.

### Electroretinography (ERG)

Briefly, mice were dark-adapted overnight and anesthetized with 1% pentobarbital sodium. Pupils were dilated with 1–2 drops of Tropicamide Phenylephrine Eye Drops and the cornea was lubricated with Hypromellose GEL. The ERG was recorded using a Diagnosys Celeris rodent ERG device. Electrodes were placed on top of each cornea. For dark-adapted ERG, mice were stimulated by flash light varying in intensity from 0.003 to 10 cd s/m^2^. The light-adapted ERG was performed following dark-adapted ERG. After a 10-min light adaption (30 cd/m^2^, White-6500 K), photopic ERG was performed with flash varying from 0.3 to 30 cd s/m^2^. Analysis of a-wave and b-wave amplitudes was performed using customized Espion ERG Data Analyzer software (version 6.63.26) that digitally filters out high-frequency oscillatory potential wavelets. The a-wave amplitude was measured from the baseline to the negative peak and the b-wave was measured from the a-wave trough to the maximum positive peak. Three to five traces were averaged per light intensity, with both eyes recorded. The traces from each eye were taken as individual measurements. The number and eye of animals used are indicated in each of the figure legends.

### Visual behavior by optomotor test

Optomotor eye tracker system (Striatech) was used to detect the visual acuity of mice. The optomotor response detects animals by gazing at a moving environment/object and turning the head (eye movement) to stabilize the image on the retina (the optomotor reflex is responsible for eye movements with the same target). The animal’s visual abilities (visual acuity) were estimated by observing their head movement owing to adjusting the environment of movement (black and white stripes). The visual behavior of the mice was not artificially interfered. We found normal BALB/c mice did not respond well to the optomotor while C57BL/6J mice showed active head movement. Therefore, we used C57BL/6J mice (6- to 8-week-old, male) in this study. The mice were kept in a completely closed box during the whole process, providing a constant rotation speed (12°/s) and a constant contrast (99.72%) of the black and white stripes. The staircase method is used to determine the spatial frequency (cyc/deg), starting from 0.056 to 0.50 cycles per degree, and measuring the maximum rotation frequency that the mouse can pass. Check clockwise first, then counterclockwise. Four monitors were used to simulate the stripes, and we could observe them through the camera above the box. The instrument’s software uses a special algorithm to measure the scores of the mice under different parameters. The representative video is shown in Additional file 10: Fig. S10 and more recordings can be accessed at https://zenodo.org/ with #7871259.

### Reagents, sources and antibodies

The reagent, source and antibody information are listed in Table 1.

### Primers

Primers used for qPCR analysis are listed in Table 2.

### Statistical analysis

Results are expressed as mean ± SD unless otherwise indicated. GraphPad Prism 8.0.2 software (GraphPad software, Inc., La Jolla, CA) was used for statistical analysis as described within Results. The statistical analysis is performed using two-tailed, unpaired *t*-test or ANOVA as indicated in the figure legends.

## Results

### dBET6 induces BRD4 degradation in mouse retinas and cultured photoreceptor cells

**Fig. 1:**
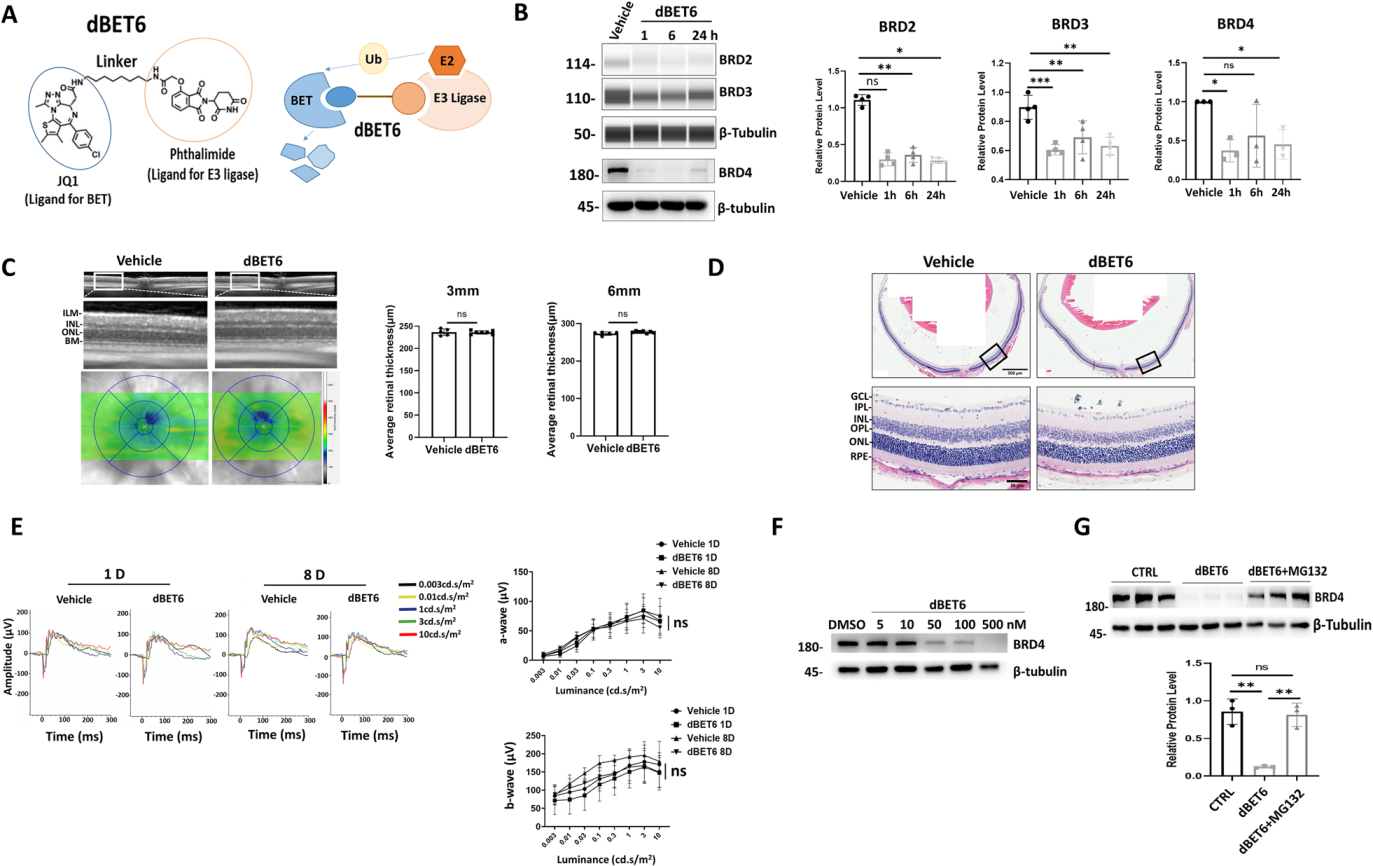
dBET6 degrades BET proteins in mouse retina and in 661W cells. **A** Diagrams show the chemical structure (left) and working model (right) of dBET6 to degrade BET proteins. **B** WES and WB analysis show degradation of BRD2, 3, and 4 in mouse retina after i.p. injection of dBET6 (10 mg/kg). Total retinal proteins were extracted at the indicated time points post-injection. WES did not give clear band for BRD4, thus traditional WB analysis was conducted for BRD4 detection. Right panels show quantification results. *ns* not significant; *p < 0.05; **p < 0.01; ***p < 0.0005, *n* = 3 eyes per group, One-way ANOVA, Tukey’s test. **C**, **D** Mice received two i.p. injections of dBET6 (10 mg/kg) with a 24 h interval. Analysis was conducted 8 days after the second injection. **C** In vivo OCT shows retina structure and heat map shows the retina thickness. The lower panels show average retinal thickness in the 3 mm and 6 mm circles. *ns* not significant, *n* = 5 mice for vehicle and *n* = 7 mice for dBET6 group, respectively. *ILM* internal limiting membrane, *INL* inner nuclear layer, *ONL* outer nuclear layer, *BM* Bruch’s membrane. **D** Representative HE staining shows retina morphology. Scale bar: 500 μm for the upper panels and 50 μm for the enlarged lower panels. *n* = 3 eyes per group. *GCL* ganglion cell layer, *IPL* inner plexiform layer, *OPL* outer plexiform layer, *RPE* retinal pigment epithelium. **E** Representative dark-adapted ERG recording. Mice received two injections of vehicle or dBET6 with a 24 h interval. Analysis was conducted 1 day (1D) or 8 days (8D) after the second injection. Right panels show luminance-response results for the a- and b-waves from mice of indicated treatment. *n* = 5 mice, 8–10 eyes per group, ns: not significant, Two-way ANOVA, Tukey’s test. **F** WB analysis show degradation of BRD4 by dBET6 treatment (2 h) in the photoreceptor-like cells 661W. **G** 661W cells were treated with DMSO or dBET6 (100 nM) for 2 h, or pretreated with MG132 (50 μM) for 2 h and then dBET6 (100 nM) was added for additional 2 h before collection for WB analysis. Right panel shows the quantification result of WB. *ns* not significant, **p < 0.01; *n* = 3 per group, one-way ANOVA, Tukey’s test

dBET6 is the second generation of BET PROTAC that selectively degrades BET proteins by the ubiquitin‒proteasome system (Fig. 1A). Firstly, we excluded the possibility that the solvent (vehicle) used to prepare dBET6 may affect retinal morphology and function (Additional file 1: Fig. S1). To determine whether dBET6 crosses the blood‒retina barrier and induces effective BET degradation, we I.P. injected dBET6 and the mouse retinas were subjected to WB or WES analysis, an automatic immunoassay. At a dose of 10 mg/kg, single injection of dBET6 induced prominent degradation of BRD2, 3 and 4 as early as 1 h post-injection, and the effect persisted for 24 h (Fig. 1B). In an 7-day observation, degradation of BRD4 lasted for 1 day, with recover began at day 2 after two dBET6 injections, which were performed in the following experiments (Additional file 2: Fig. S2). dBET6 did not affect retinal structure or function, as determined by OCT, HE staining and ERG analysis 8 days after injection (Fig. 1C–E, Additional file 3: Fig. S3). At 1 day after the second injection, comparable ERG responses were observed in both the vehicle and dBET6-injected mice, hence excluding short-term effects of dBET6 (Fig. 1E, Additional file 3: Fig. S3). Furthermore, dBET6 did not affect the body weight or structure of the major organs, although slightly increased spleen size was detected (Additional file 4: Fig. S4). Next, the effect of dBET6 was evaluated on cultured retinal cells. dBET6 treatment led to decreased BRD4 protein levels in the photoreceptor-like cell line 661W in a dose-dependent manner (Fig. 1F), while the addition of the proteasome inhibitor MG132 reversed dBET6-induced BRD4 degradation (Fig. 1G). Together, these results indicated that systemic application of dBET6 efficiently degraded BET proteins in mouse retina, and confirmed that dBET6-induced BRD4 degradation through the ubiquitin–proteasome system.

### dBET6 ameliorates retinal response impairment after LD

**Fig. 2:**
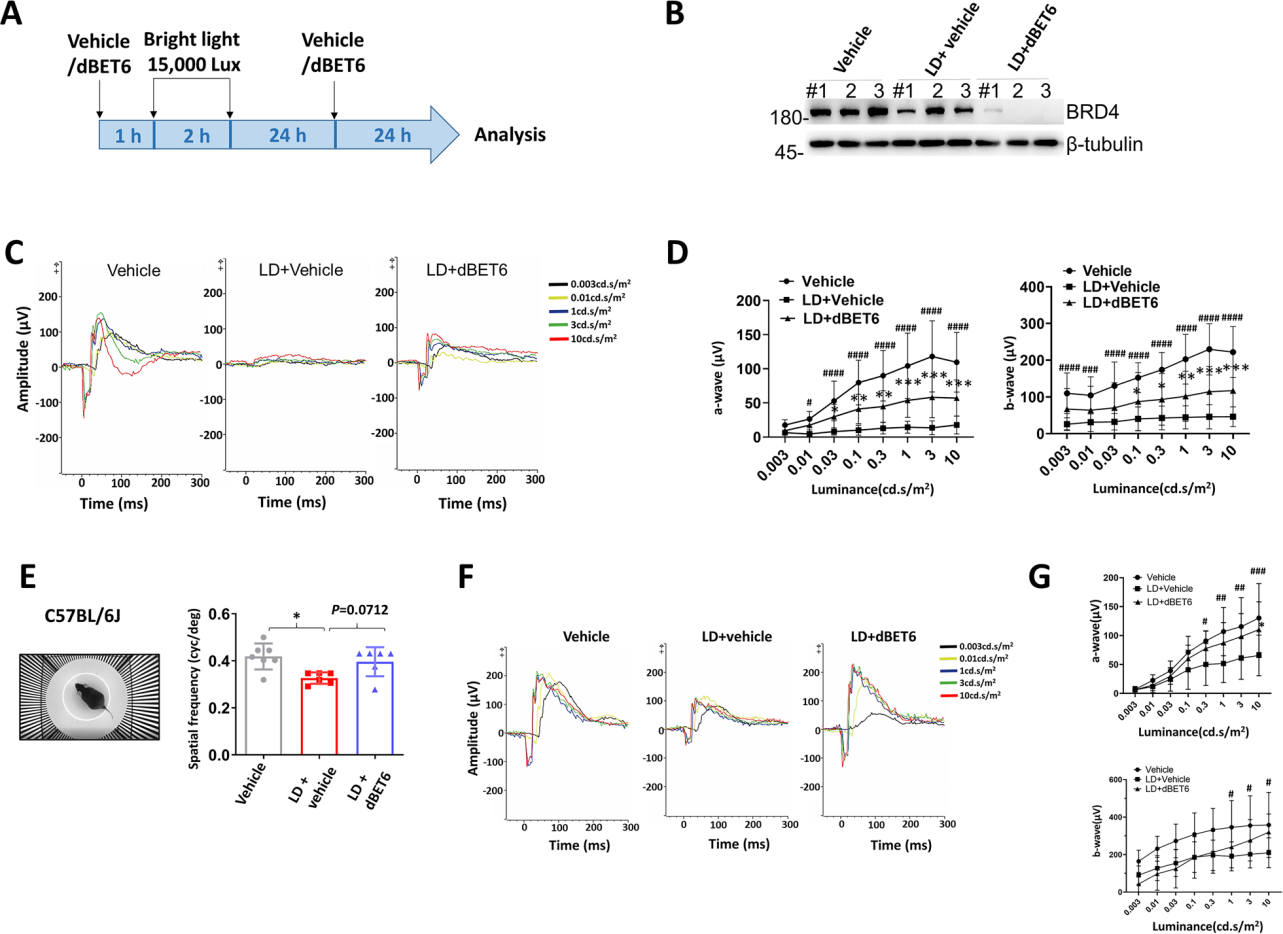
dBET6 inhibits LD-induced retinal function impairment. **A** Schematic illustration shows the experimental design. Mice were i.p. injected with dBET6 (10 mg/kg) at 1 h before and 24 h after 2-h light exposure. WB analysis (**B**) and visual tests (**C**–**G**) were performed at 48 h post-LD. For mouse strain: **B**–**D**, BALB/cJ; **E**–**G**, C57BL/6J. **B** WB analysis shows degradation of BRD4 protein in mouse retinas with dBET6 treatment. **C** Representative dark-adapted ERG results. **D** Luminance-response results for the a- and b-waves. *n* = 8–10 mice, 16–20 eyes per group. *ns* not significant, # or *p < 0.05; ## or **p < 0.01; ### or ***p < 0.0005, ####: *p* < 0.0001 (#: Vehicle versus LD + Vehicle, *: LD + Vehicle versus LD + dBET6), two-way ANOVA, Tukey’s test. **E** Left: a captured image to illustrate the optomotor apparatus used in the study. The C57BL/6J mice were used in this study as described in the methods. Right: visual acuity measured as the spatial frequency threshold in mice of indicated treatment. *: *p* < 0.05, *n* = 6–7 mice per group, One-way ANOVA, Tukey’s test. **F** Representative dark-adapted ERG results performed on C57BL/6J mice. **G** Quantification results of a- and b-waves with indicated treatment on C57BL/6J mice. *n* = 4 mice, 6–8 eyes per group. # or *: *p* < 0.05; ##: *p* < 0.01; ###: *p* < 0.0005 (#: Vehicle versus LD + Vehicle, *: LD + Vehicle versus LD + dBET6), two-way ANOVA, Tukey’s test

Next, we investigated the effects of dBET6 on retinal function after LD (Fig. 2A), and the effect of dBET6 on the degradation of BRD4 was confirmed in light-exposed retinas (Fig. 2B). dBET6 significantly alleviated LD-induced retinal impairment as indicated by dark-adapted ERG analysis (Fig. 2C, D). The ERG a- and b-wave amplitudes were greater in dBET6-injected mice than in the vehicle-injected mice after LD (Fig. 2C, D), indicating protection of dBET6 on photoreceptor phototransduction (a-wave) and rod bipolar cell’ depolarization (b-wave). However, dBET6 did not show significant effect on light-adapted ERG, suggesting losses in cone function was not prevented by dBET6 (Additional file 5: Fig. S5A, B). We then compared the effect of single dBET6 injected either 1 h prior to or 24 h after LD. ERG, OCT and HE analysis indicated that pretreatment with dBET6 1 h before LD was necessary for retinal protection, whereas dBET6 injection 24 h post-LD showed no evident protection (Additional file 6: Fig. S6). Next, the visual acuity was determined by an optomotor response test (Fig. 2E). Visual acuity was quantified by increasing spatial frequency of full contrast gratings until no optokinetic response could be determined. We found normal BALB/c mice did not respond well to the optomotor, C57BL/6J mice were thus used in this study. As shown in Fig. 2E, LD led to lower visual acuity, in contrast, dBET6 treatment improved, although not significantly, visual acuity. ERG analysis confirmed that the retinal response in C57BL/6J mice was impaired after LD, and dBET6 mitigated dark-adapted ERG a-wave and light-adapted ERG b-wave reduction (Fig. 2F, G, Additional file 5: Fig. S5C, D). In addition, TUNEL staining revealed LD-induced photoreceptor death in the ONL, which was reversed by dBET6 treatment (Additional file 7: Fig. S7A). dBET6 also inhibited activation of macrophages/microglia after LD, as evidenced by reduced IBA1 and CD86-positive cells in the retina (Additional file 7: Fig. S7B, C). However, as previously reported [32], C57BL/6J mice showed greater resistance to LD than the BALB/c strain, as revealed by the absence of evident retinal degeneration (Additional file 7: Fig. S7D, E). Taken together, our results indicate that dBET6 treatment alleviates LD-induced retinal responsiveness loss.

### dBET6 reduces retinal degeneration induced by LD

**Fig. 3:**
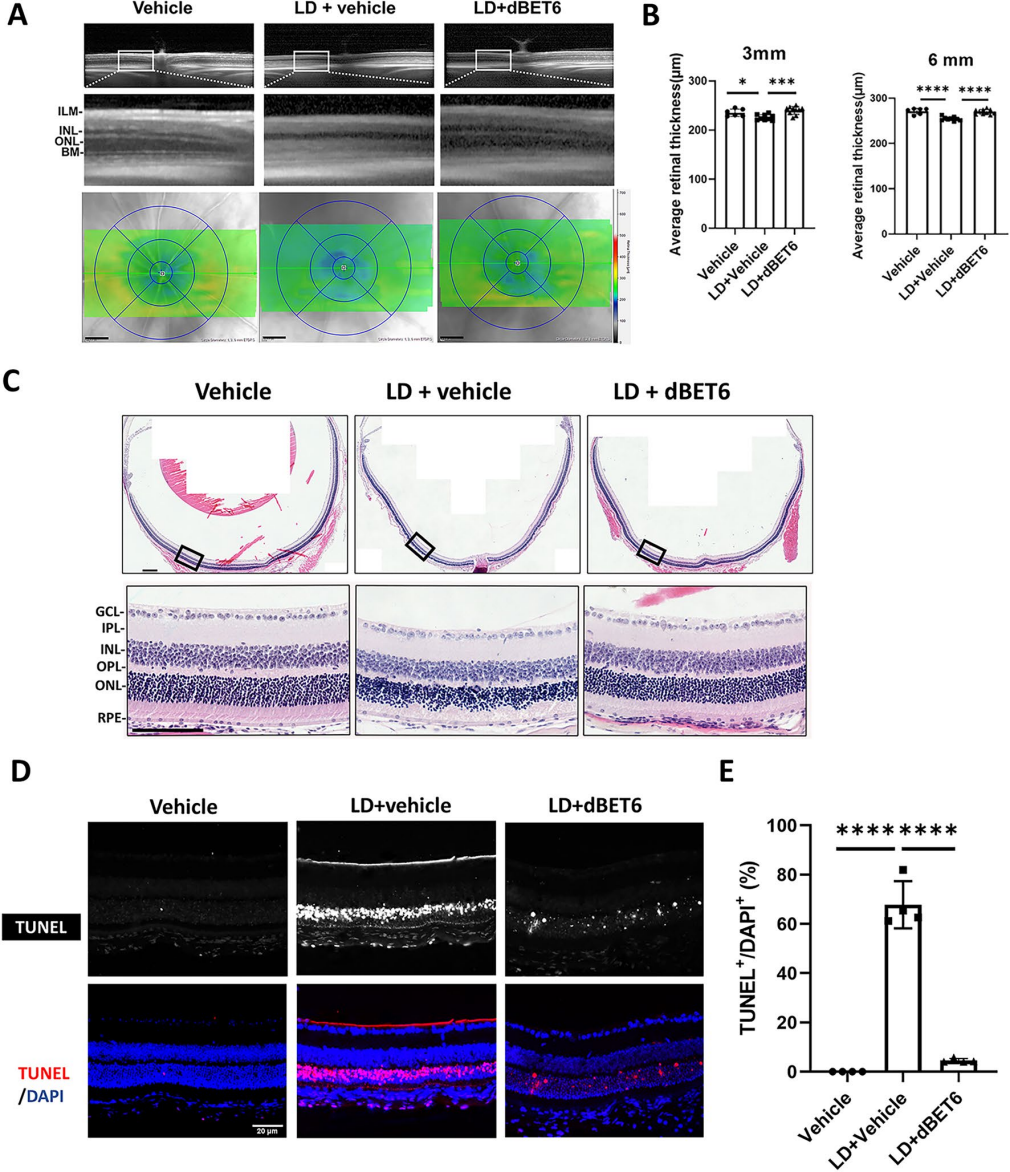
Administration of dBET6 alleviates LD-induced retina degeneration. **A** In vivo OCT shows retinal thickness and lamination with indicated treatment. LD led to altered reflectance in the ONL, which was reversed by dBET6 treatment. Lower panel: heat maps show the average retinal thickness. Scale bar: 100 μm. **B** Quantification of retinal thickness in the 3 mm and 6 mm circles. *: *p* < 0.05, ***: *p* < 0.0005, ****: *p* < 0.0001, one-way ANOVA, Tukey’s test; *n* = 6 mice for vehicle, *n* = 5 mice for LD + vehicle or LD + dBET6. **C** HE staining shows retinal structure with indicated treatment. Scale bar: 100 μm. *n* = 3 eyes for each group. **D** TUNEL staining shows retinal cell death with indicated treatment. Upper panel: TUNEL signals were indicated by white fluorescence. Lower panel: overlapping images of TUNEL and DAPI (nuclei). Scale bar: 20 μm. **E** Quantification of TUNEL-positive retinal cells in ONL of indicated treatment. For each group, four regions were randomly selected and the percentage of TUNEL-positive cells were calculated over DAPI-positive cells within ONL. ****: *p* < 0.0001, *n* = 3 eyes for each group, One-way ANOVA, Tukey’s test

The improvement in retinal function after dBET6 treatment suggests that the retinal structure and photoreceptors may be protected from LD. Indeed, in vivo OCT imaging, which detects changes in outer nuclear layer (ONL) reflectance, showed that the dBET6-treated mice exhibited reduced hyperreflective photoreceptor layer compared to vehicle-treated mice after LD (Fig. 3A, upper panels). Volume scans revealed thinning of the retina in vehicle-treated mice after LD, which was rescued in the dBET6-treated groups (Fig. 3A, lower panels and Fig. 3B). HE staining further confirmed the protective effect of dBET6 on the overall retinal structure after LD (Fig. 3C). The retinal photoreceptor death is a hallmark for LD. As shown in Fig. 3D, TUNEL staining indicated evident photoreceptor death in the ONL, which was significantly reversed by dBET6 treatment (Fig. 3D, E). Taken together, these results demonstrate that dBET6 inhibits LD-induced retinal degeneration and photoreceptor death.

### dBET6 reduces macrophages/microglia reactivity and reactive gliosis in response to LD

**Fig. 4:**
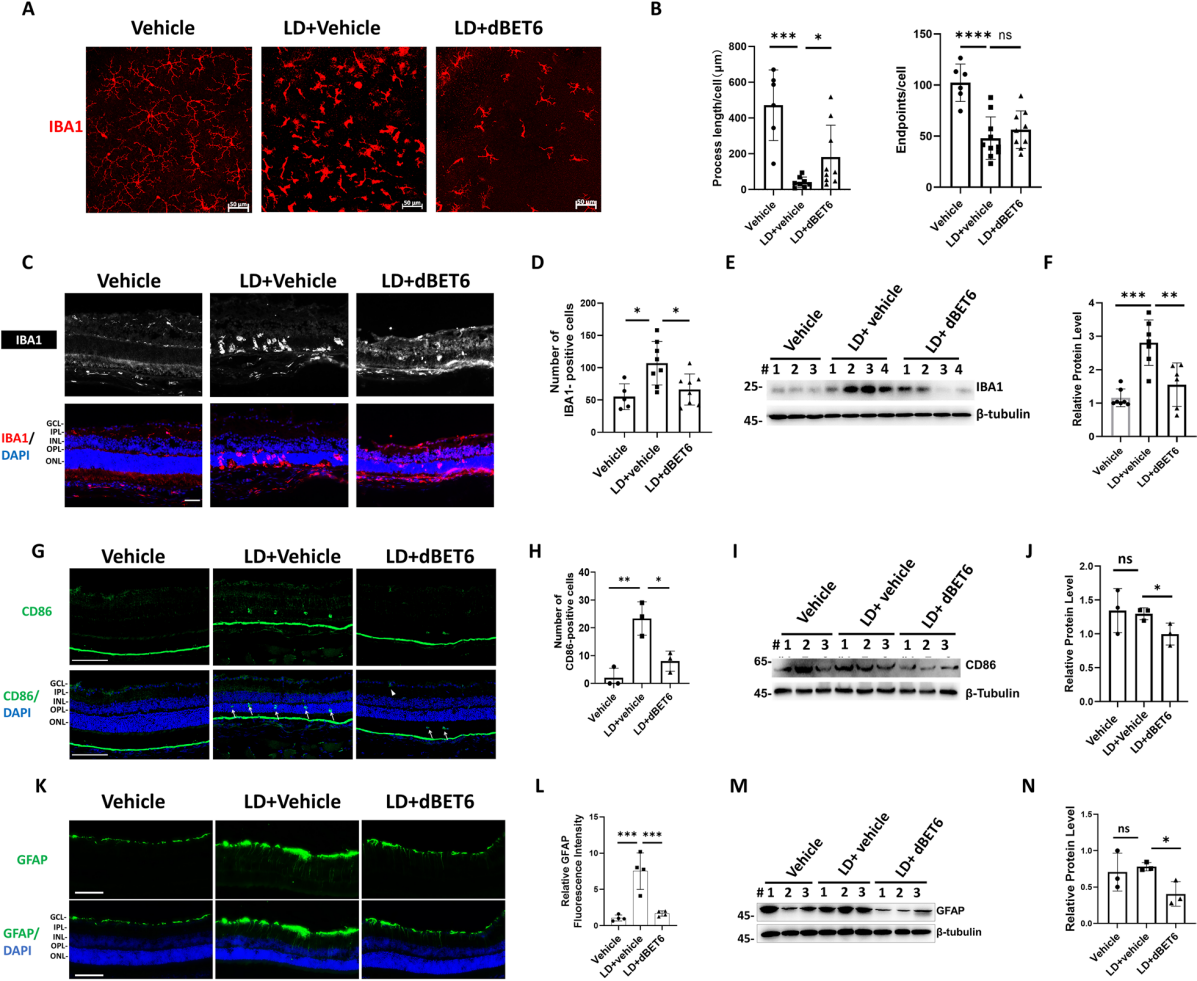
dBET6 inhibits LD-induced microglia activation and gliosis. **A** IF analysis shows morphology of IBA1-labeled microglia in retinal flat mounts. **B** Quantification results of mean microglia processes and endpoints. For each group, six to ten regions were randomly selected in the whole mounts, total length of the branches and the total number of endpoints in the region were measured by Image J and divided by the IBA1-positive cell number. *ns* not significant, *: *p* < 0.05, ***: *p* < 0.0005, ****: *p* < 0.0001, *n* = 3 eyes per group, One-way ANOVA, Tukey’s test. For vehicle treatment, ~ 50 cells were measured, for for LD + vehicle or LD + dBET6, 130–200 cells were measured. **C** IF analysis shows IBA1-positive cells in mouse retina with indicated treatment. The macrophages/microglia were immune labeled by anti-IBA1 antibody. Scale bar: 50 μm. **D** Quantification results of IBA1-positive cells. For each treatment, five to eight regions of the whole retinas were randomly selected and the IBA1-positive cells were counted. For vehicle treatment, *n* = 3 eyes; for LD + vehicle or LD + dBET6, *n* = 4 eyes per group, respectively. *: *p* < 0.05, One-way ANOVA, Tukey’s test. **E** Representative WB analysis shows IBA1 protein levels with indicated treatment. Total retinal proteins were extracted and subject to WB analysis. **F** Quantification of WB analysis. **p < 0.01; ***p < 0.0005, *n* = 6–8 eyes per group, One-way ANOVA, Tukey’s test. **G**. IF analysis shows active macrophages/microglia labeled by anti-CD86. Arrows show CD86-positive cells infiltrating photoreceptor after LD. Arrow heads show CD86-positive cells in the GCL after LD. Scale bar: 100 μm. **H** Quantification results of CD86-positive cells. Two to three regions were randomly selected in the whole retina and the CD86-positive cells were counted. *: *p* < 0.05, **: *p* < 0.01, *n* = 3 eyes per group, One-way ANOVA, Tukey’s test. **I** WB analysis shows CD86 protein levels with indicated treatment. Total retinal proteins were extracted and subject to WB analysis. **J** Quantification of WB analysis. *ns* not significant, *: *p* < 0.05, *n* = 3 eyes per group, One-way ANOVA, Tukey’s test. **K** IF analysis shows GFAP signal in mouse retina with indicated treatment. Scale bar: 100 μm. **L** Quantification of GFAP fluorescence intensity. Four regions were randomly selected in the whole retina and the GFAP fluorescence intensity were measured. ***: *p* < 0.0005, *n* = 3 eyes for each treatment group, One-way ANOVA, Tukey’s test. **M** WB analysis shows GFAP protein levels with indicated treatment. Total retinal proteins were extracted and subject to WB analysis. **N** Quantification of WB analysis. *ns* not significant, *: *p* < 0.05, *n* = 3 eyes for each treatment group, One-way ANOVA, Tukey’s test

Retinal macrophages/microglia activation is a shared feature of retinal inflammation and contributes to retinal degeneration. We determined the effects of dBET6 on macrophages/microglia reactivity in response to LD. In control mice, macrophages/microglia immunolabeled by IBA1 showed a typical ramified resting phenotype in the retinal flat mounts (Fig. 4A). LD led to presence of amoeboid-shaped macrophages/microglia, characterized by significantly reduced process length and endpoints (Fig. 4A, B). Injection of dBET6 partially inhibited the reactive microglia/macrophages phenotype following LD, evidenced by increased cell process and increased, although not significantly, process endpoints (Fig. 4A, B). In addition, resting microglia/macrophages are distributed in the inner and outer plexiform layers, LD led to infiltration of reactive macrophages/microglia in the degenerating photoreceptor layer and the subretinal area, which was remarkably inhibited by dBET6 treatment (Fig. 4C, D). WB analysis further confirmed that dBET6 repressed LD-induced IBA1 protein expression (Fig. 4E, F). CD86 is a commonly used marker for active microglia/macrophages [33]. Similar to IBA1 staining results, LD led to infiltration of CD86-positive cells in the photoreceptor layer (arrows), which was inhibited by dBET6 treatment (Fig. 4G, H), and WB also indicated that dBET6 suppressed retinal CD86 protein level after LD (Fig. 4I, J). To better address the reactive phenotype of glial cells, glial fibrillary acid protein (GFAP), which is a widely used marker of Müller cell and astrocyte reactivity, was investigated by immunostaining. In control retina, GFAP was confined to the ganglion cell layer (GCL) as expected, while LD induced increased GFAP signal in the inner plexiform layer (IPL) and inner nuclear layer (INL) (Fig. 4K, L). dBET6 suppressed LD-induced GFAP expression, which was confirmed by WB analysis (Fig. 4M, N). Finally, to determine if dBET6 is toxic to microglia/macrophages, we compared cell viability in mice retina without light exposure. TUNEL labeling at 24 h after dBET6 injection did not reveal cell death in IBA1-positive cells (Additional file 8: Fig. S8A). However, the dBET6-injected retina showed a decrease in CD86 protein (Additional file 8: Fig. S8B, C), but there was no significant change in *Cd86* RNA expression after dBET6 treatment in control mice as shown in RNA-seq (Additional file 8: Fig. S8D). Currently we do not know the mechanism for dBET6-mediated CD86 downregulation. Nevertheless, these results indicated that dBET6 effectively inhibited LD-induced macrophages/microglia activation and gliosis in the mouse retina.

### dBET6 inhibits LPS-induced microglia activation

**Fig. 5:**
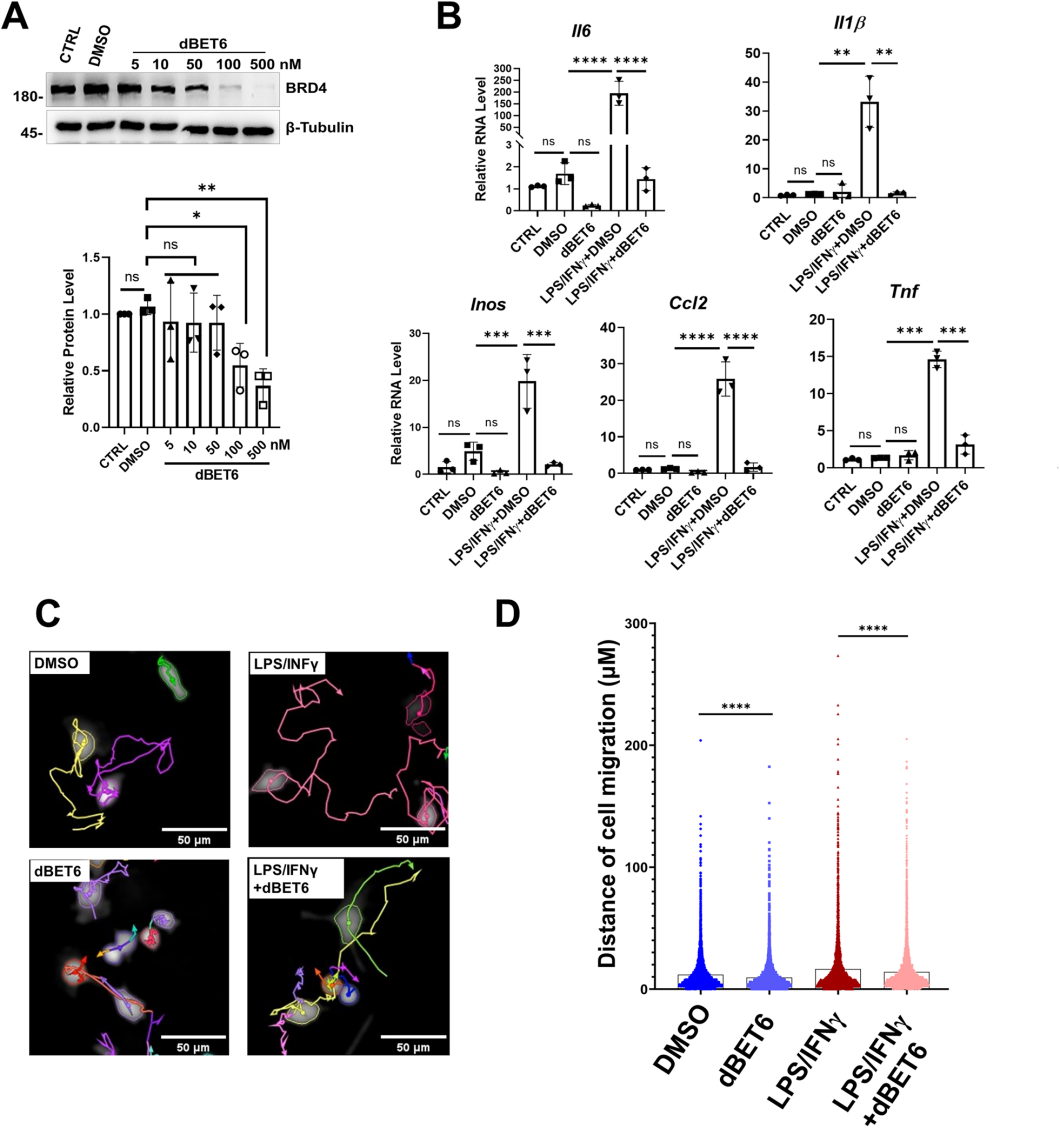
dBET6 inhibits LPS-induced microglia activation and migration. **A** WB analysis shows the indicated protein expression. BV2 cells were treated without (CTRL) or with dBET6 at the indicated concentration for 2 h and then total proteins were extracted for WB analysis. The lower panel shows quantification result of WB. *ns* not significant, *: *p* < 0.05, One-way ANOVA, Tukey’s test, *n* = 3 per group. **B** qRT-PCR analysis shows the indicated gene expression. BV2 Cells were left untreated (CTRL) or treated with the indicated drugs for 24 h before analysis. For LPS/ IFNγ, combined LPS (10 ng/ml) and IFN-γ (20 ng/ml) were used. For dBET6 treatment, 100 nM dBET6 was used. *ns* not significant, **: *p* < 0.01, ***: *p* < 0.0005, ***: *p* < 0.0001. One-way ANOVA, Tukey’s test, *n* = 3 per group. **C** High-content image analysis was used to access BV2 motility with the indicated treatment. Cell mobility was recorded in live BV2 cells for 8 h. Each cell migration route was labeled. Drug concentrations were the same as described in **B**. **D** Quantification results of cell migration. For each group, at least 300 cells were recorded. ****: *p* < 0.0001. One-way ANOVA, Tukey’s test

Reactive microglia produce pro-inflammatory factors which exacerbate retina degeneration. We directly assessed the effects of dBET6 on BV2 cells, a widely used mouse brain microglial cell line, in response to inflammatory stimulation. WB analysis showed that 50 nM dBET6 degraded BRD4 (Fig. 5A). We used LPS and IFNγ (LPS/IFNγ) to polarize the BV2 cell into proinflammatory state [34]. BET6 remarkably repressed LPS/IFNγ-induced proinflammatory factor expression, such as *IL1β*, *TNF* and *IL6* (Fig. 5B). Increased cell migration is a typical feature of microglial activation. Therefore, we determined BV2 cell mobility by high-content live cell analysis, which can record real-time cell migration (Fig. 5C). dBET6 significantly inhibited cell migration in the presence or absence of LPS/IFNγ (Fig. 5D). Together, these results showed that dBET6 efficiently reduced microglial reactivity in response to LPS treatment.

### LD led to activation of cGAS-STING innate immunity

**Fig. 6:**
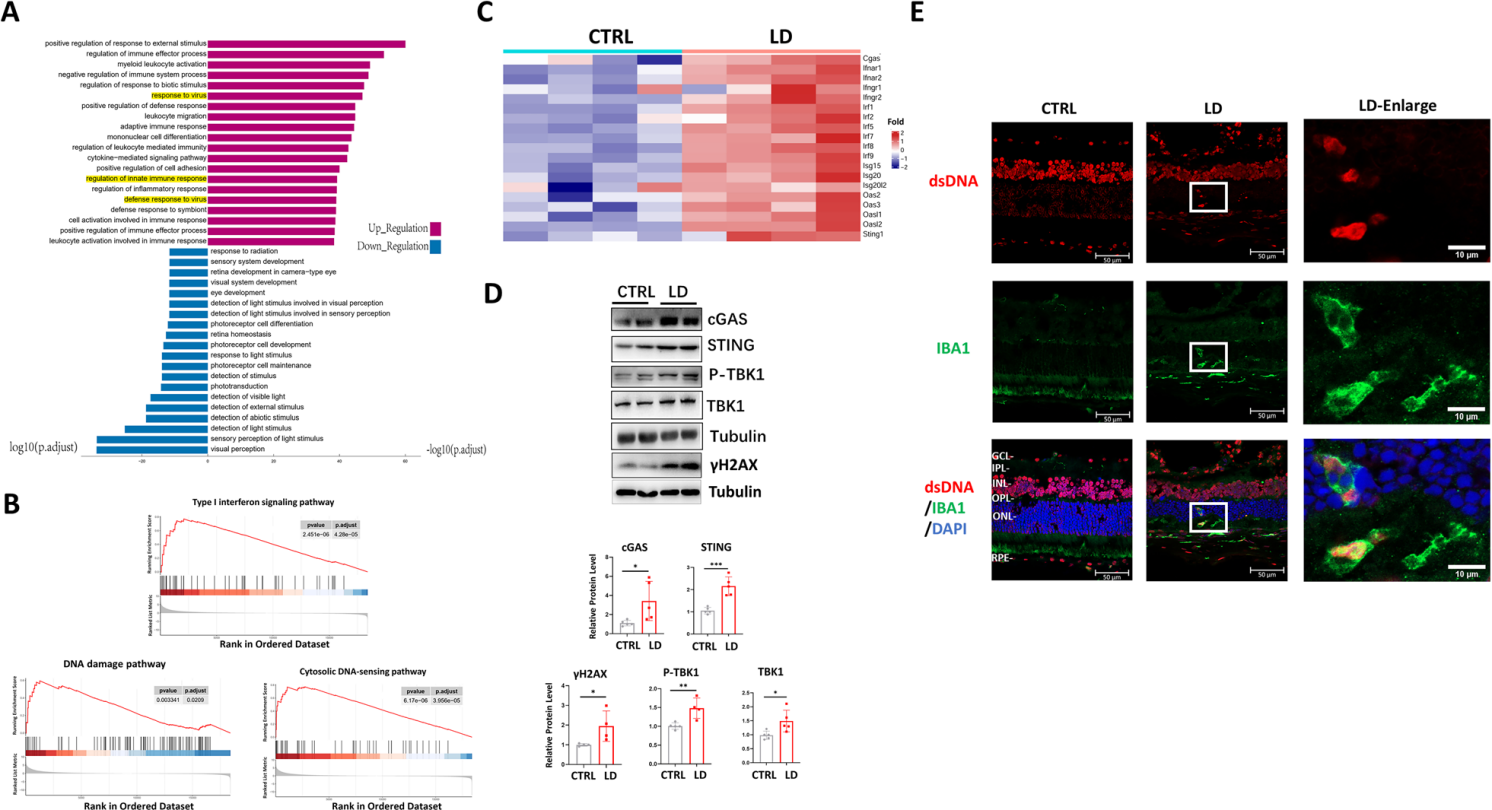
LD led to activation of cGAS-STING innate immunity in mouse retina. **A**–**D** Mice were expose with or without bright light and retinas were collected 48 h after exposure. Total RNAs or proteins were extracted and subjected to RNA-sequencing or WB analysis or IHC analysis, respectively. *n* = 4 for each group. **A** Gene Ontology (GO) analysis shows significant up- or down-regulated biological processes. Numbers indicate log10 and − log10 of the *p*. adjust. **B** Gene set enrichment analysis (GSEA) profiles demonstrate significant enrichment of gene sets associated with indicated pathway in light-exposed mouse retinas compared to control retinas. **C** Heat map shows increased cGAS-STING genes in LD group as compared with CTRL. *p* < 0.05. **D** WB analysis shows indicated protein level in mouse retinas. Right panels show the quantification results of the presented WB and a repeated WB not shown. *: *p* < 0.05, **: *p* < 0.01, ***: *p* < 0.001, *n* = 4 per group, unpaired *t*-test. **E** The DNA was labeled by anti-dsDNA antibody. Note that the cytosolic DNA seems to be engulfed by IBA1-positive microglia/macrophages in the ONL. Scale bar: 50 and 10 μm

Our recent results showed robust activation of cGAS-STING innate immune signaling in retinal after SI treatment [13]. Although both SI and LD led to retina degeneration, SI primarily affected RPE while LD induced photoreceptor death. Therefore, to determine transcriptome alterations, we performed RNA-seq analysis of control and LD-exposed mouse retinas. LD activated inflammatory responses, the top upregulated pathways include immune effector process, leukocyte activation and migration, cytokine signaling and innate immune response (Fig. 6A). In contrast, genes involved in the visual system, light stimulus, photoreceptor differentiation and development were severely repressed, confirming the deleterious effects of LD on retinal neurons (Fig. 6A). Intriguingly, we found that genes enriched in cytosolic DNA sensing and the type I interferon pathway were significantly upregulated (Fig. 6B). Specifically, the upregulation of *cGAS*, *STING*, and downstream IRF genes, as well as interferon-stimulated genes (*Isg* and *Oas* genes), were induced by LD (Fig. 6C). We further determined cGAS-STING pathway protein expression, and WB analysis showed that LD led to the significant upregulation of cGAS and STING protein levels and activation/phosphorylation of downstream TBK1 (Fig. 6D). We also observed a remarkable DNA damage response after LD, as evidenced by the increased DNA damage marker γH2Ax (Fig. 6D). In line with activation of cGAS-STING, LD led to accumulation of cytosolic DNA in the photoreceptors (Fig. 6E), and macrophages/microglia were recruited to lesions with cytosolic DNA leakage (Fig. 6E, enlarged region). These results indicate that LDs induces cytosolic accumulation and activation of cGAS-STING-mediated innate immunity in the mouse retina.

### dBET6 inhibits LD-induced cGAS-STING activation in mouse retina

**Fig. 7:**
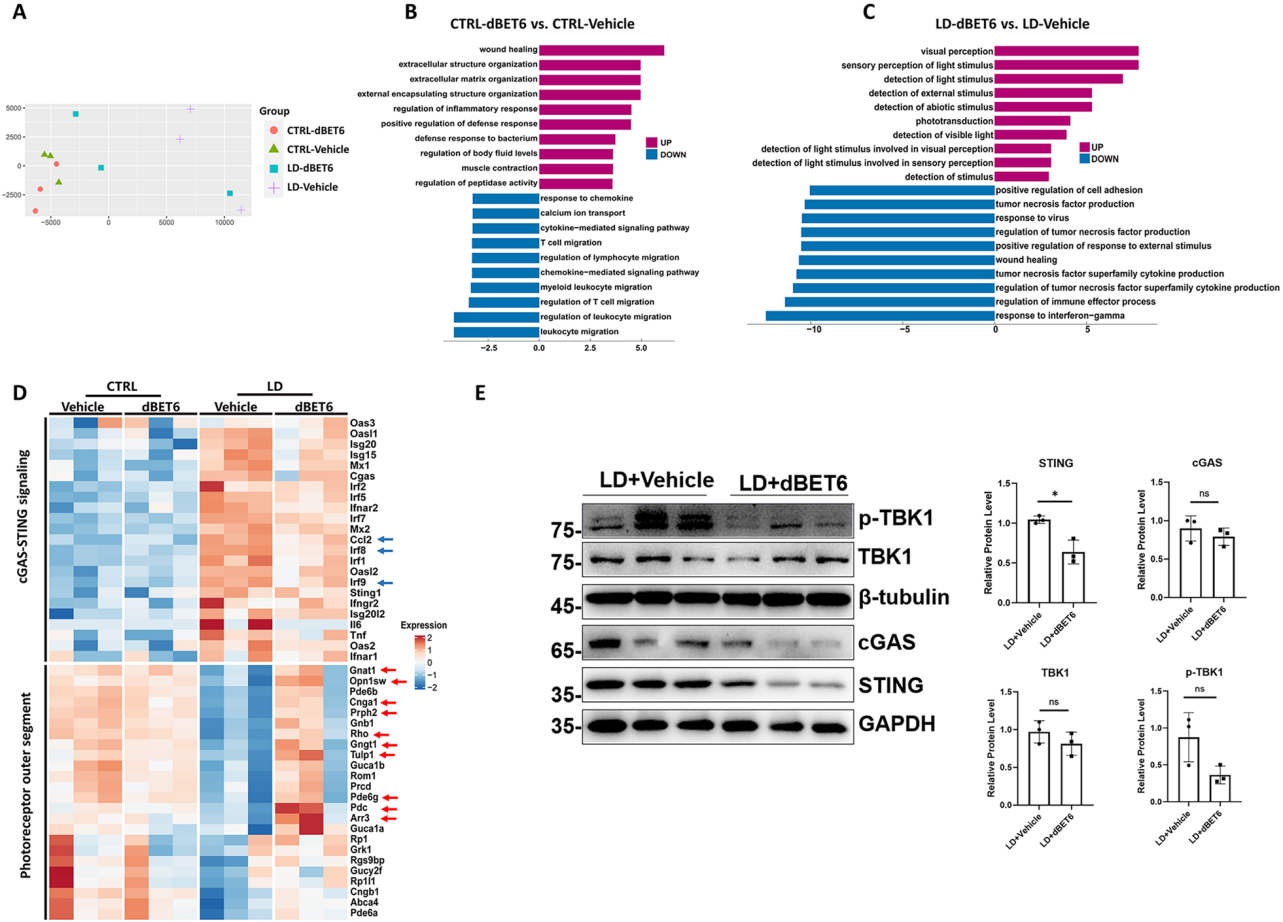
dBET6 suppresses LD-induced retinal photoreceptor gene loss and inflammatory response. **A** Multi-dimensional scaling (MDS) plot shows variation among 12 retinal RNA-Seq samples, distance between sample labels indicates dissimilarity. **B**, **C** GO analysis shows significant up- or down-regulated biological processes in control (**B**) or LD (**C**) mice. Numbers indicate log10 and − log10 of the *p*. adjust. Fold change ≥ 1.5. **D** Heat map shows genes involved in cGAS-STING and photoreceptor outer segment in the indicated treatment groups. Arrows show genes with ≥ 1.5 fold change, *p* < 0.05. **E** WB analysis shows indicated protein level in mouse retinas. Right panels show quantification results of WB. *ns* not significant, *: *p* < 0.05, *n* = 3 eyes per group, unpaired *t*-test

To determine the impact of dBET6 on retinal transcriptome, we performed RNA-seq in control or LD mouse retinas, with or without dBET6 treatment. Multi-dimensional scaling (MDS) revealed similar gene expression profile between vehicle and dBET6 groups in control mice (Fig. 7A). However, in the presence of LD, two of the dBET6 samples showed a distinct expression profile compared to vehicle samples (Fig. 7A). Gene ontology (GO) analysis showed dBET6 treatment led to downregulation of genes involved in immune cell migration in control retina (Fig. 7B). More importantly, dBET6 preserved genes responsible for visual function and reduced genes involved in cell chemotaxis, cytokine production and response to interferon in LD retinas (Fig. 7C). Heatmap further illustrated that dBET6 partially reversed LD-induced cGAS-STING activation, and inhibited photoreceptor genes loss in response to LD (Fig. 7D), supporting a retinal protection of dBET6 at the molecular level. Furthermore, genes preserved by dBET6 after LD are mostly involved in rod phototransduction pathway, such as *Gnat1*, *Cnga1*, *Rhodopsin*, *Gngt1*, *Pde6g* and *Pdc* (Fig. 7D). This may lead to greater protection of the dark-adapted than light-adapted ERG response by dBET6 (compare Fig. 2C, D and Additional file 5: Fig. S5A, B). Finally, the inhibitory effects of dBET6 on the protein levels of cGAS-STING components were determined by WB analysis, and STING was significantly decreased by dBET6 (Fig. 7E).

### dBET6 represses LD-induced cGAS-STING signaling in reactive macrophages/microglia

**Fig. 8:**
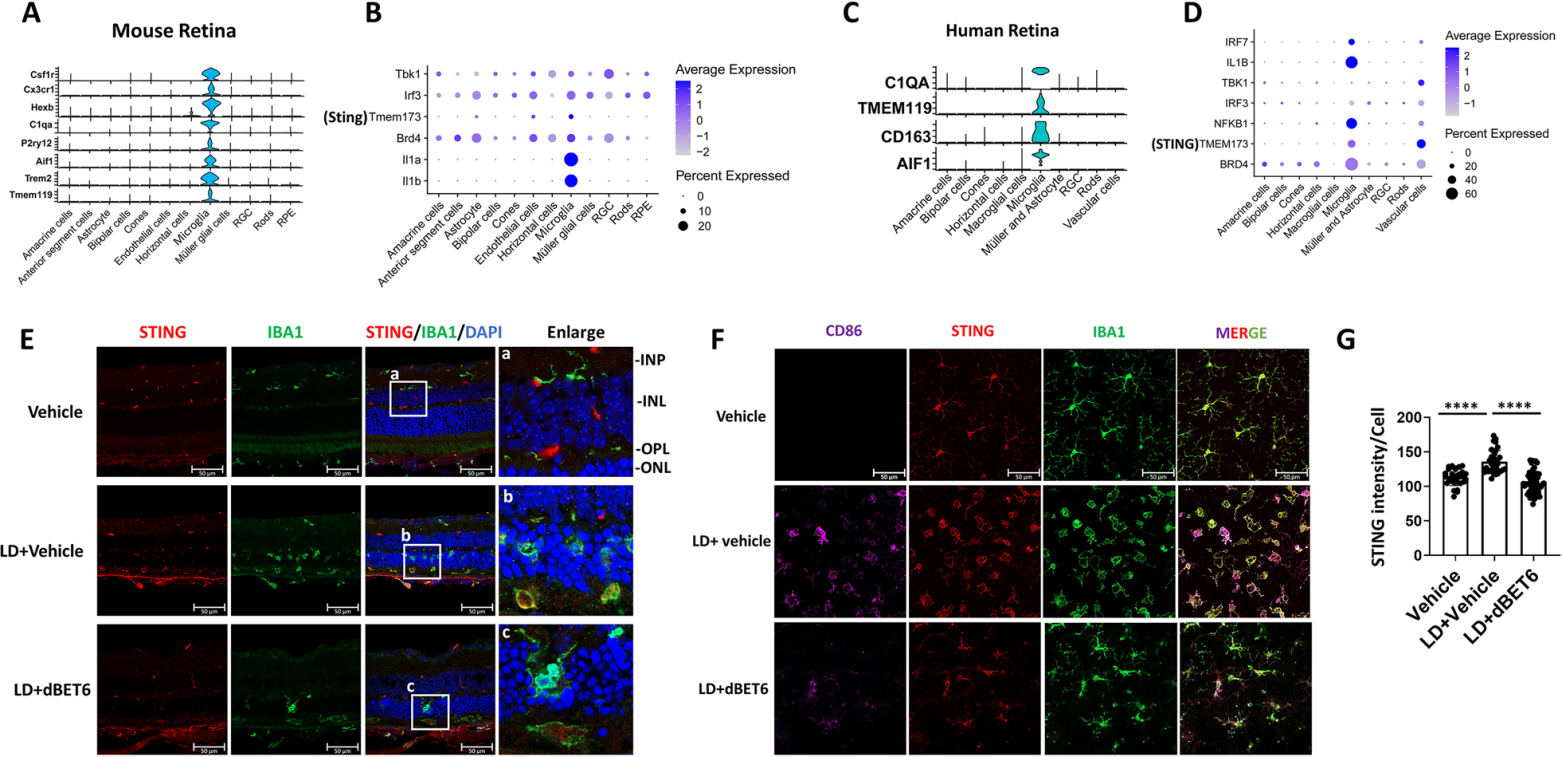
dBET6 treatment inhibits STING expression in microglia/macrophages in response to LD. **A**, **C** Average expression of known microglia marker genes are used to identify the mouse (**A**) and human (**C**) retinal microglia cell cluster. Violin plots of genes previously reported to be enriched in microglia cell populations are plotted (cellmarker2.0, http://yikedaxue.slwshop.cn/). Mouse sc-RNA data are extracted from https://github.com/jiewwwang/Single-cell-retinal-regeneration, and human sc-RNA data can be accessed by GEO#: GSE137537. **B**, **D** Dot plot of cGAS-STING signaling genes and BRD4 in retinal microglia. **E** IHC shows indicated protein expression in mouse retina. Note STING-positive cells were localized to the GCL, IPL and INL in control mice. LD led to infiltration of IBA1-positive macrophages/microglia to the photoreceptors, and most of these infiltrating/reactive mononuclear phagocyte show STING staining (enlarged figure b). Administration of dBET6 reduced IBA1-positive cell in the photoreceptors and STING signal (enlarged figure c). Scale bar: 50 μm. D. IF analysis of retinal flat mounts. Note the evident overlapping of STING and IBA1 in both ramified (resting) and amoeboid-shaped (reactive) microglia/macrophages. Scale bar: 50 μm. **E** Quantification of STING IF intensity from the retinal flat mounts. For each group, six to ten regions were randomly selected in the whole mounts, the intensity of STING signal was measured by Image J. About 30 cells measured for Vehicle or LD + Vehicle, and ~ 60 cells measured for LD + dBET6. ****: *p* < 0.0001, One-way ANOVA, Tukey’s test, *n* = 3 retinas per group

Next, we sought to determine the retinal cell type(s) that express STING and the cell-specific effects of dBET6 on STING. We first analyzed published mouse and human retinal scRNA-seq databases [29, 35] (Fig. 8A, C). In mouse retina, we found that *Sting* was enriched in microglia, and also expressed in endothelial cells and astrocytes (Fig. 8B). *Irf3* and *Tbk1* were expressed in all retinal cell types; however, RGC and microglia showed higher levels of *Tbk1*, whereas astrocytes, endothelial cells, microglia and RPE exhibited higher levels of *Irf3* (Fig. 8B). Interestingly, the expression of *Brd4* was comparatively high in microglia (Fig. 8B). In human retina, *IRF7* was mainly expressed in microglia, while *TBK1* was enriched in vascular cells (Fig. 8D). *STING* was detected in both microglia and vascular cells, with a higher level observed in vascular cells (Fig. 8D). Furthermore, although BRD4 was expressed in most human retinal cell populations, highest expression percentage was detected in microglia (Fig. 8D). Taken together, these findings suggest that dBET6 may inhibit STING by degrading BRD4 in microglia. We then performed IHC analysis on retinal cryosections. In control retinas, STING-positive cells were mainly observed in the inner and outer plexiform layers, some were found adjacent to the process of IBA1-positive microglia/macrophages (Fig. 8E-a). After LD, most microglia/macrophages infiltrating the photoreceptor layer exhibited positive STING staining (Fig. 8E-b). Administration of dBET6 repressed the accumulation of microglia/macrophages in photoreceptors and inhibited STING expression in infiltrated microglia/macrophages (Fig. 8E-c). To improve the visualization of STING signal in microglia/macrophages, IHC was then performed on retinal flat mounts. Compared to cryosections, we observed a clear overlapping of STING and IBA1 in resting microglia from the flat mounts (Fig. 8F). Similarly, LD induced amoeboid phenotype in reactive microglia/macrophages, where STING co-localized with CD86 and IBA1 (Fig. 8F). Treatment with dBET6 inhibited microglia/macrophages activation and reduced levels of STING signal in these cells (Fig. 8F, G). Together, our results demonstrated that STING is expressed in both mouse and human retinal microglia, and that dBET6 effectively repressed STING levels in mouse microglia/macrophages in response to LD.

## Discussion

**Fig. 9:**
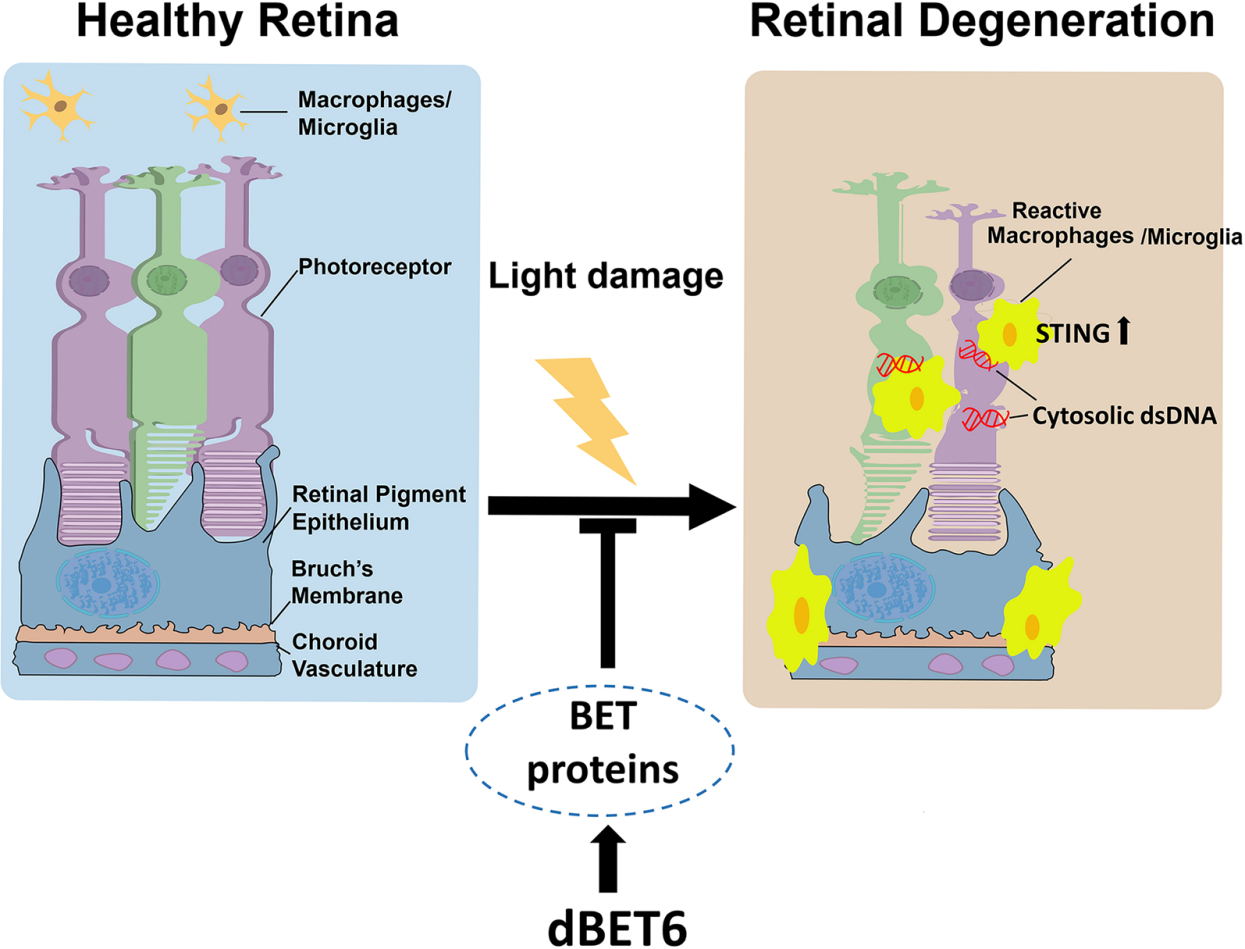
Schematic model for dBET6 protection against LD-induced retinal degeneration. During LD, retinal macrophages/microglia were transited from resting state to activated from, migrating into photoreceptor layer. Cytosolic DNA was accumulated in photoreceptors and cGAS-STING signaling was activated in retina. Reactive macrophages/microglia were recruited to photoreceptors with DNA leakage, and macrophages/microglia there exhibited increased STING expression. Degradation of BET proteins inhibited LD-induced macrophages/microglia activation and STING expression

In this study, we present three novel findings. First, the PROTAC molecule dBET6 efficiently and rapidly degrades BET proteins in mouse retinas. Second, dBET6 improves photoreceptor viability and retinal function after LD. Third, dBET6 represses LD-induced retinal inflammation and STING activation in microglia/macrophages (Fig. 9). To our knowledge, this is the first evidence showing the protective effects and detailed cellular/molecular mechanism of BET PROTAC in retinal degeneration.

By hijacking the ubiquitin‒proteasome system, BET PROTACs selectively and completely induce the degradation of BET proteins within the nanomolar range, making them a promising strategy for treating inflammatory disorders and cancer. Recent studies have reported that dBET1 reduces acute ischemic brain injury and neuroinflammation [36, 37]. Here, we used dBET6, which shows tenfold increased efficiency in degrading BRD4 by increasing cell permeability [18]. One issue to be considered is whether dBET6 can exert toxicity due to increased potency. However, our cellular and organ analyses did not show detectable toxicity of dBET6. Furthermore, although BET PROTACs induce cancer cell death and xenograft tumor growth arrest, it was reported to not affect mouse body weight or normal tissues [38, 39]. However, extended observation times following dBET6 administration would be necessary to better evaluate its safety.

Persistent inflammation has been recognized as a major pathogenic factor for retinal degeneration. Accordingly, drugs that inhibit inflammation and reduce the activity of microglia/macrophages in the retia have shown promising therapeutic potential in diverse retinal degeneration models [5–7]. cGAS-STING signaling is a key cytosolic DNA-sensing innate immunity pathway that regulates inflammation, cell apoptosis and reactive oxygen production [40, 41]. Our previous study revealed damaged DNA leaking into the cytosol of photoreceptors and the activation of cGAS-STING in response to sodium iodate exposure. Here, cytosolic DNA and activation of cGAS-STING signaling was detected after LD. These results suggest that cGAS-STING may be a common immune response mechanism associated with retinal damage. Furthermore, we found that STING was upregulated in microglia/macrophages infiltrating the photoreceptor after LD. Interestingly, during the preparation of the manuscript, Ma et al. reported elevated cGAS and STING expression in retinal myeloid cells in oxygen-induced retinopathy (OIR), and knockout or inhibition of STING alleviated microglial activation in OIR [42]. Therefore, considering our scRNA-seq findings that cGAS-STING components are enriched in retinal microglia/macrophages, suppressing cGAS-STING in retinal microglia/macrophages cells may be beneficial in treating retinal degenerative diseases and retinopathy. Future investigations using myeloid cell-specific STING knockout mice may substantiate this hypothesis.

Our study found that LD led to release of cytosolic dsDNA in photoreceptors, the primary cell affected by bright light. It is possible that following DNA leakage, the damaged photoreceptor produces interferon and other inflammatory molecules, which led to infiltration and activation of microglia/macrophages to ONL. Once recruited, these microglia/macrophages may phagocyte damaged photoreceptors and their cytosolic DNA, thereby activating and amplifying cGAS-STING signaling and retinal inflammation. Treatment with dBET6 reduces microglia migration and cGAS-STING activation, mitigating retinal inflammation. However, microglia can also be neuroprotective as depletion of microglia has been reported accelerates photoreceptor death in response to LD or retinal detachment [43, 44]. The retinal protective function of active microglia may be due to its communication with other immune cells such as macrophages, or phagocytosis of dead photoreceptor [44]. In addition, microglia/macrophages can be activated and polarized into metabolically and functionally distinct subpopulations, i.e., proinflammatory and anti-inflammatory polarization states [45]. Currently, the impact of dBET6 and cGAS-STING on microglia/macrophages phagocytosis activity and polarity is unknown. Given *STING* is enriched in mouse and human microglia, we are in the process of investigating the microglia subpopulation alteration in STING-depleted mouse retina.

## Conclusions

In summary, we have shown for the first time that BET PROTAC dBET6 rapidly degrades BET protein in the retina, and exerts potent protective effects against retinal degeneration in response to LD. Our results have also revealed global activation of cGAS-STING signaling, and upregulation of STING in the reactive macrophages/microglia during pathogenesis of LD-induced retinal degeneration. dBET6 reduced the LD-induced STING expression in macrophages/microglia, repressed retinal inflammation and photoreceptor death, suggesting targeted BET protein degradation a potential therapeutic option for retinal degenerative diseases.

## Supplementary Information

**Additional file 1: Figure S1.** Retinal morphology and function in control and vehicle-injected mice. Mice were i.p. injected withoutor with vehiclefor two times, with a 24-h interval. The indicated analysis was performed 24 h after the second injection. **A** OCT analysis shows in vivo retina morphology. **B** Quantification of the retinal thickness in 3 mm and 6 mm circles in the OCT images. *n* = 3 mice per group, *ns* not significant, unpaired *t*-test. **C** HE staining shows retinal structure. Scale bar: 200 µm for the upper panels and 50 µm for the lower panels. *n* = 3 eyes per group. **D**, **E** Representative ERG analysis shows retinal response. *n* = 4 mice, 8 eyes per group.

**Additional file 2: Figure S2.** WB analysis shows retinal BRD4 protein levels. **A** Schematic diagram shows experimental procedure. For day 0, one i.p. injection of dBET6 was performed and the retinal proteins were collected 24 h post-injection. For other groups, two injections of dBET6 or vehicle were administered and the retinal proteins were extracted at the indicated time points. For vehicle injection, proteins were collected at day 7. **B** WB analysis of indicated protein. Right panel: quantification of WB. *n* = 4 eyes per group. **p < 0.01, ***p < 0.0005, One-way ANOVA, Tukey’s test, comparison made between dBET6 samples with Vehicle.

**Additional file 3: Figure S3.** Photopic ERG recording of vehicle and dBET6-injected mouse retinas. **A** Representative light-adapted ERG recording. BALB/cJ mice received two injections of vehicle or dBET6 with a 24 h interval. Analysis was conducted 1 dayor 8 daysafter the second injection. **B** Luminance-response results for the b-waves from mice of indicated treatment. *n* = 5 mice, 8–10 eyes per group, *ns* not significant, Two-way ANOVA, Tukey’s test.

**Additional file 4: Figure S4.** Systemic administration of dBET6 did not cause obvious organ destruction. I.p. injections of vehicleor dBET6were performed on the 1st and 2nd day of experiment, with 24 h interval. **A** Mouse body weight beforeand 8 daysafter injections. No significant differences were observed between the vehicle- and dBET6-injected groups. **B** Left panel: the indicated organs are shown before collection for HE staining 8 days after injection. Right panel: quantification results of the tissue area. The tissue area was calculated by Image J. *n* = 3 for CTRL and *n* = 5 for dBET6 treatment. *ns* not significant, *: *p* < 0.05, unpaired *t*-test. **C** HE staining of the indicated organs. Scale bar: 20 μm.

**Additional file 5: Figure S5.** Photopic ERG recordings of mouse retinas with or without dBET6 treatment. Mice were treated as described in Fig. 2A. **A**, **C** Representative light-adapted ERG results in BALB/cJand C57BL/6J. **B**, **D** Luminance-response results for the b-waves in BALB/cJand C57BL/6J. *ns* not significant, #*p* < 0.05; ## or **p < 0.01; ###*p* < 0.0005, #### or****: *p* < 0.0001, two-way ANOVA, Tukey’s test. *n* = 8–10 mice, 16–20 eyes per group for BALB/cJ; *n* = 4 mice, 6–8 eyes per group for C57BL/6J.

**Additional file 6: Figure S6.** Retinal morphology and function in mice injected with dBET6 1 h prior to or 24 h after LD. **A** Schematic diagram shows experimental design. One injection of dBET6was administered at either 1 h before or 24 h after LD. The analysis was performed 48 h post-LD. For LD + vehicle group, vehicle was injected 1 h before LD. **B** OCT analysis shows in vivo retina morphology. **C** Quantification of the retinal thickness in 3 mm and 6 mm circles in the OCT images. *n* = 6 eyes per group, *ns* not significant, *: *p* < 0.05, **: *p* < 0.01, One-way ANOVA, Tukey’s test. **D** HE staining shows retinal structure. Scale bar: 200 µm for the upper panels and 50 µm for the lower panels. *n* = 3 eyes per group. **E**, **F**. Representative dark-adaptedand light-adaptedERG recording shows retinal response. *n* = 4 mice, 6–8 eyes per group.

**Additional file 7: Figure S7.** Retinal morphology and microglia/macrophages reactivity in C57BL/6J mice. **A** TUNEL analysis shows cell death in the indicated treatment. *n* = 3 eyes per group, scale bar: 50 μm. **B** IF analysis shows IBA1-positive microglia/macrophages in mouse retinas. *n* = 3 eyes per group, scale bar: 100 μm. **C** IF analysis using retinal flat mounts. The reactive microglia/macrophages were labeled by anti-CD86 antibody. *n* = 3 eyes per group, scale bar: 50 μm. **D** OCT analysis shows in vivo retina morphology. Lower panels: quantification of the retinal thickness in 3 mm and 6 mm circles in the OCT images. *n* = 4 eyes for vehicle and *n* = 5 eyes for LD + vehicle or LD + dBET6. No significant retinal thickness alteration was detected between each group. **E** HE staining shows retinal structure. Scale bar: 200 µm for the upper panels and 50 µm for the lower panels. *n* = 3 eyes per group.

**Additional file 8: Figure S8.** dBET6 treatment did not cause retinal microglia/macrophages cell death but reduced CD86 protein level. Two injections of vehicle or dBET6 were performed with a 24 h interval. The indicated analysis was conducted at 24 h after the second injection. **A** IF analysis using retinal flat mounts. The microglia/macrophages were labeled by anti-IBA1 antibody. Note not detectable cell death existed in vehicle or dBET6 treatment, as indicated by TUNEL staining. *n* = 3 eyes per group, scale bar: 20 μm. **B** WB analysis of the indicated proteins. **C** Quantification of WB results. *ns* not significant, *: *p* < 0.05, unpaired *t*-test, *n* = 3 eyes per group. **D** RNA-seq result of the indicated genes. The RNA-seq was conducted as described in Fig. 7. *ns* not significant, **: *p* < 0.01, ****: *p* < 0.0001.

**Additional file 9: Figure S9.** Original WB images in this study. The uncropped WB, and the images merged with the protein marker are shown.

**Additional file 10: Figure S10.** Representative video for visual behavior test.

## Data Availability

All are included in the article and Additional files 1, 2, 3, 4, 5, 6, 7, 8, 9, 10.

## References

[1] Pfau M, von der Emde L, de Sisternes L, Hallak JA, Leng T, Schmitz-Valckenberg S, Holz FG, Fleckenstein M, Rubin DL (2020). Progression of photoreceptor degeneration in geographic atrophy secondary to age-related macular degeneration. JAMA Ophthalmol.

[2] Wright AF, Chakarova CF, Abd El-Aziz MM, Bhattacharya SS (2010). Photoreceptor degeneration: genetic and mechanistic dissection of a complex trait. Nat Rev Genet.

[3] Zhao L, Zabel MK, Wang X, Ma W, Shah P, Fariss RN, Qian H, Parkhurst CN, Gan WB, Wong WT (2015). Microglial phagocytosis of living photoreceptors contributes to inherited retinal degeneration. EMBO Mol Med.

[4] Hume DA, Perry VH, Gordon S (1983). Immunohistochemical localization of a macrophage-specific antigen in developing mouse retina: phagocytosis of dying neurons and differentiation of microglial cells to form a regular array in the plexiform layers. J Cell Biol.

[5] Scholz R, Sobotka M, Caramoy A, Stempfl T, Moehle C, Langmann T (2015). Minocycline counter-regulates pro-inflammatory microglia responses in the retina and protects from degeneration. J Neuroinflamm.

[6] Fernando N, Natoli R, Valter K, Provis J, Rutar M (2016). The broad-spectrum chemokine inhibitor NR58-3.14.3 modulates macrophage-mediated inflammation in the diseased retina. J Neuroinflamm.

[7] Wang X, Zhao L, Zhang Y, Ma W, Gonzalez SR, Fan J, Kretschmer F, Badea TC, Qian HH, Wong WT (2017). Tamoxifen provides structural and functional rescue in murine models of photoreceptor degeneration. J Neurosci.

[8] Belkina AC, Nikolajczyk BS, Denis GV (2013). BET protein function is required for inflammation: Brd2 genetic disruption and BET inhibitor JQ1 impair mouse macrophage inflammatory responses. J Immunol.

[9] Marmorstein R, Zhou MM (2014). Writers and readers of histone acetylation: structure, mechanism, and inhibition. Cold Spring Harb Perspect Biol.

[10] Shang E, Salazar G, Crowley TE, Wang X, Lopez RA, Wang X, Wolgemuth DJ (2004). Identification of unique, differentiation stage-specific patterns of expression of the bromodomain-containing genes Brd2, Brd3, Brd4, and Brdt in the mouse testis. Gene Expr Patterns.

[11] Zhao L, Li J, Fu Y, Zhang M, Wang B, Ouellette J, Shahi PK, Pattnaik BR, Watters JJ, Wong WT, Guo LW (2017). Photoreceptor protection via blockade of BET epigenetic readers in a murine model of inherited retinal degeneration. J Neuroinflamm.

[12] Liu L, Yang C, Candelario-Jalil E (2021). Role of BET proteins in inflammation and CNS diseases. Front Mol Biosci.

[13] Zou M, Ke Q, Nie Q, Qi R, Zhu X, Liu W, Hu X, Sun Q, Fu JL, Tang X (2022). Inhibition of cGAS-STING by JQ1 alleviates oxidative stress-induced retina inflammation and degeneration. Cell Death Differ.

[14] Filippakopoulos P, Qi J, Picaud S, Shen Y, Smith WB, Fedorov O, Morse EM, Keates T, Hickman TT, Felletar I (2010). Selective inhibition of BET bromodomains. Nature.

[15] Lu J, Qian Y, Altieri M, Dong H, Wang J, Raina K, Hines J, Winkler JD, Crew AP, Coleman K, Crews CM (2015). Hijacking the E3 ubiquitin ligase cereblon to efficiently target BRD4. Chem Biol.

[16] Zhou XL, Zhao F, Xu YT, Guan YY, Yu T, Zhang YZ, Duan YC, Zhao Y (2022). A comprehensive review of BET-targeting PROTACs for cancer therapy. Bioorg Med Chem.

[17] Cochran AG, Conery AR, Sims RJ (2019). Bromodomains: a new target class for drug development. Nat Rev Drug Discov.

[18] Winter GE, Mayer A, Buckley DL, Erb MA, Roderick JE, Vittori S, Reyes JM, di Iulio J, Souza A, Ott CJ (2017). BET bromodomain proteins function as master transcription elongation factors independent of CDK9 recruitment. Mol Cell.

[19] Sun L, Wu J, Du F, Chen X, Chen ZJ (2013). Cyclic GMP-AMP synthase is a cytosolic DNA sensor that activates the type I interferon pathway. Science.

[20] Ishikawa H, Barber GN (2008). STING is an endoplasmic reticulum adaptor that facilitates innate immune signalling. Nature.

[21] Motwani M, Pesiridis S, Fitzgerald KA (2019). DNA sensing by the cGAS-STING pathway in health and disease. Nat Rev Genet.

[22] Wu J, Sun L, Chen X, Du F, Shi H, Chen C, Chen ZJ (2013). Cyclic GMP-AMP is an endogenous second messenger in innate immune signaling by cytosolic DNA. Science.

[23] Chen Q, Sun L, Chen ZJ (2016). Regulation and function of the cGAS-STING pathway of cytosolic DNA sensing. Nat Immunol.

[24] Fremond ML, Crow YJ (2021). STING-mediated lung inflammation and beyond. J Clin Immunol.

[25] Hinkle JT, Patel J, Panicker N, Karuppagounder SS, Biswas D, Belingon B, Chen R, Brahmachari S, Pletnikova O, Troncoso JC (2022). STING mediates neurodegeneration and neuroinflammation in nigrostriatal alpha-synucleinopathy. Proc Natl Acad Sci USA.

[26] Jin M, Shiwaku H, Tanaka H, Obita T, Ohuchi S, Yoshioka Y, Jin X, Kondo K, Fujita K, Homma H (2021). Tau activates microglia via the PQBP1-cGAS-STING pathway to promote brain inflammation. Nat Commun.

[27] Zou M, Gong L, Ke Q, Qi R, Zhu X, Liu W, Sun Q, Tang X, Luo Z, Gong X (2022). Heterochromatin inhibits cGAS and STING during oxidative stress-induced retinal pigment epithelium and retina degeneration. Free Radic Biol Med.

[28] Kerur N, Fukuda S, Banerjee D, Kim Y, Fu D, Apicella I, Varshney A, Yasuma R, Fowler BJ, Baghdasaryan E (2018). cGAS drives noncanonical-inflammasome activation in age-related macular degeneration. Nat Med.

[29] Hoang T, Wang J, Boyd P, Wang F, Santiago C, Jiang L, Yoo S, Lahne M, Todd LJ, Jia M (2020). Gene regulatory networks controlling vertebrate retinal regeneration. Science.

[30] Young K, Morrison H (2018). Quantifying microglia morphology from photomicrographs of immunohistochemistry prepared tissue using ImageJ. J Vis Exp.

[31] Zhang X, Lan Y, Xu J, Quan F, Zhao E, Deng C, Luo T, Xu L, Liao G, Yan M (2019). Cell Marker: a manually curated resource of cell markers in human and mouse. Nucleic Acids Res.

[32] Wenzel A, Grimm C, Samardzija M, Reme CE (2005). Molecular mechanisms of light-induced photoreceptor apoptosis and neuroprotection for retinal degeneration. Prog Retin Eye Res.

[33] Jurga AM, Paleczna M, Kuter KZ (2020). Overview of general and discriminating markers of differential microglia phenotypes. Front Cell Neurosci.

[34] He L, Jhong JH, Chen Q, Huang KY, Strittmatter K, Kreuzer J, DeRan M, Wu X, Lee TY, Slavov N (2021). Global characterization of macrophage polarization mechanisms and identification of M2-type polarization inhibitors. Cell Rep.

[35] Menon M, Mohammadi S, Davila-Velderrain J, Goods BA, Cadwell TD, Xing Y, Stemmer-Rachamimov A, Shalek AK, Love JC, Kellis M, Hafler BP (2019). Single-cell transcriptomic atlas of the human retina identifies cell types associated with age-related macular degeneration. Nat Commun.

[36] DeMars KM, Yang C, Candelario-Jalil E (2019). Neuroprotective effects of targeting BET proteins for degradation with dBET1 in aged mice subjected to ischemic stroke. Neurochem Int.

[37] Liu L, Yang C, Lavayen BP, Tishko RJ, Larochelle J, Candelario-Jalil E (2022). Targeted BRD4 protein degradation by dBET1 ameliorates acute ischemic brain injury and improves functional outcomes associated with reduced neuroinflammation and oxidative stress and preservation of blood–brain barrier integrity. J Neuroinflamm.

[38] Shi C, Zhang H, Wang P, Wang K, Xu D, Wang H, Yin L, Zhang S, Zhang Y (2019). PROTAC induced-BET protein degradation exhibits potent anti-osteosarcoma activity by triggering apoptosis. Cell Death Dis.

[39] Qin AC, Jin H, Song Y, Gao Y, Chen YF, Zhou LN, Wang SS, Lu XS (2020). The therapeutic effect of the BRD4-degrading PROTAC A1874 in human colon cancer cells. Cell Death Dis.

[40] Hayman TJ, Baro M, MacNeil T, Phoomak C, Aung TN, Cui W, Leach K, Iyer R, Challa S, Sandoval-Schaefer T (2021). STING enhances cell death through regulation of reactive oxygen species and DNA damage. Nat Commun.

[41] Decout A, Katz JD, Venkatraman S, Ablasser A (2021). The cGAS-STING pathway as a therapeutic target in inflammatory diseases. Nat Rev Immunol.

[42] Ma X, Wu W, Liang W, Takahashi Y, Cai J, Ma JX (2022). Modulation of cGAS-STING signaling by PPARalpha in a mouse model of ischemia-induced retinopathy. Proc Natl Acad Sci USA.

[43] O’Koren EG, Yu C, Klingeborn M, Wong AYW, Prigge CL, Mathew R, Kalnitsky J, Msallam RA, Silvin A, Kay JN (2019). Microglial function is distinct in different anatomical locations during retinal homeostasis and degeneration. Immunity.

[44] Okunuki Y, Mukai R, Pearsall EA, Klokman G, Husain D, Park DH, Korobkina E, Weiner HL, Butovsky O, Ksander BR (2018). Microglia inhibit photoreceptor cell death and regulate immune cell infiltration in response to retinal detachment. Proc Natl Acad Sci USA.

[45] Murray PJ (2017). Macrophage polarization. Annu Rev Physiol.

