# Supplementary figures and images for: The BET PROTAC inhibitor dBET6 protects against retinal degeneration and inhibits the cGAS-STING in response to light damage
Source: J Neuroinflammation. 2023 May 22;20:119. doi: 10.1186/s12974-023-02804-y (PMC10201800; doi:10.1186/s12974-023-02804-y)

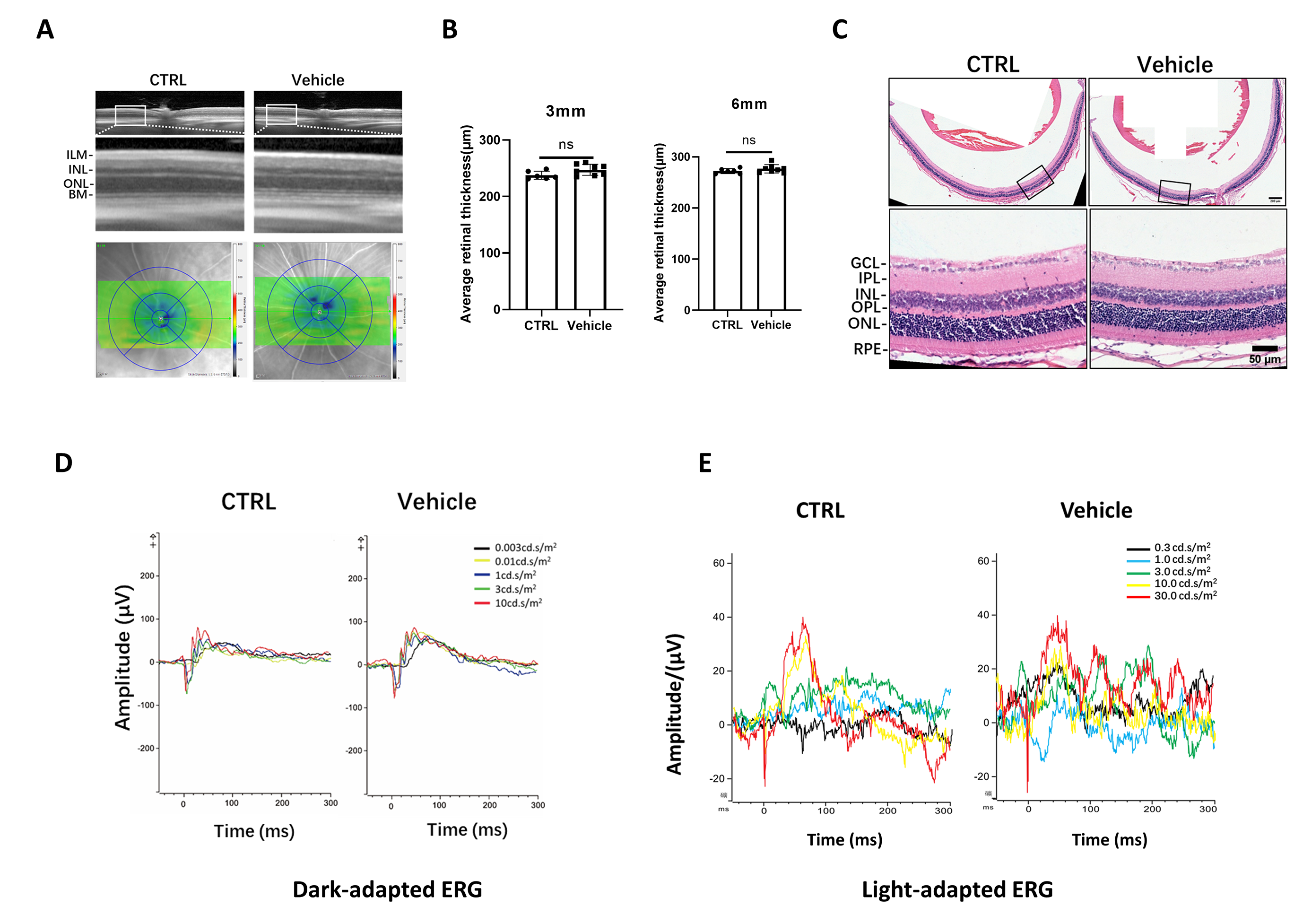

Supplement: Supplementary file 1 — Additional file 1: Figure S1. Retinal morphology and function in control and vehicle-injected mice. Mice were i.p. injected withoutor with vehiclefor two times, with a 24-h interval. The indicated analysis was performed 24 h after the second injection. A OCT analysis shows in vivo retina morphology. B Quantification of the retinal thickness in 3 mm and 6 mm circles in the OCT images. n = 3 mice per group, ns not significant, unpaired t-test. C HE staining shows retinal structure. Scale bar: 200 µm for the upper panels and 50 µm for the lower panels. n = 3 eyes per group. D, E Representative ERG analysis shows retinal response. n = 4 mice, 8 eyes per group. [file 12974_2023_2804_MOESM1_ESM.tif]

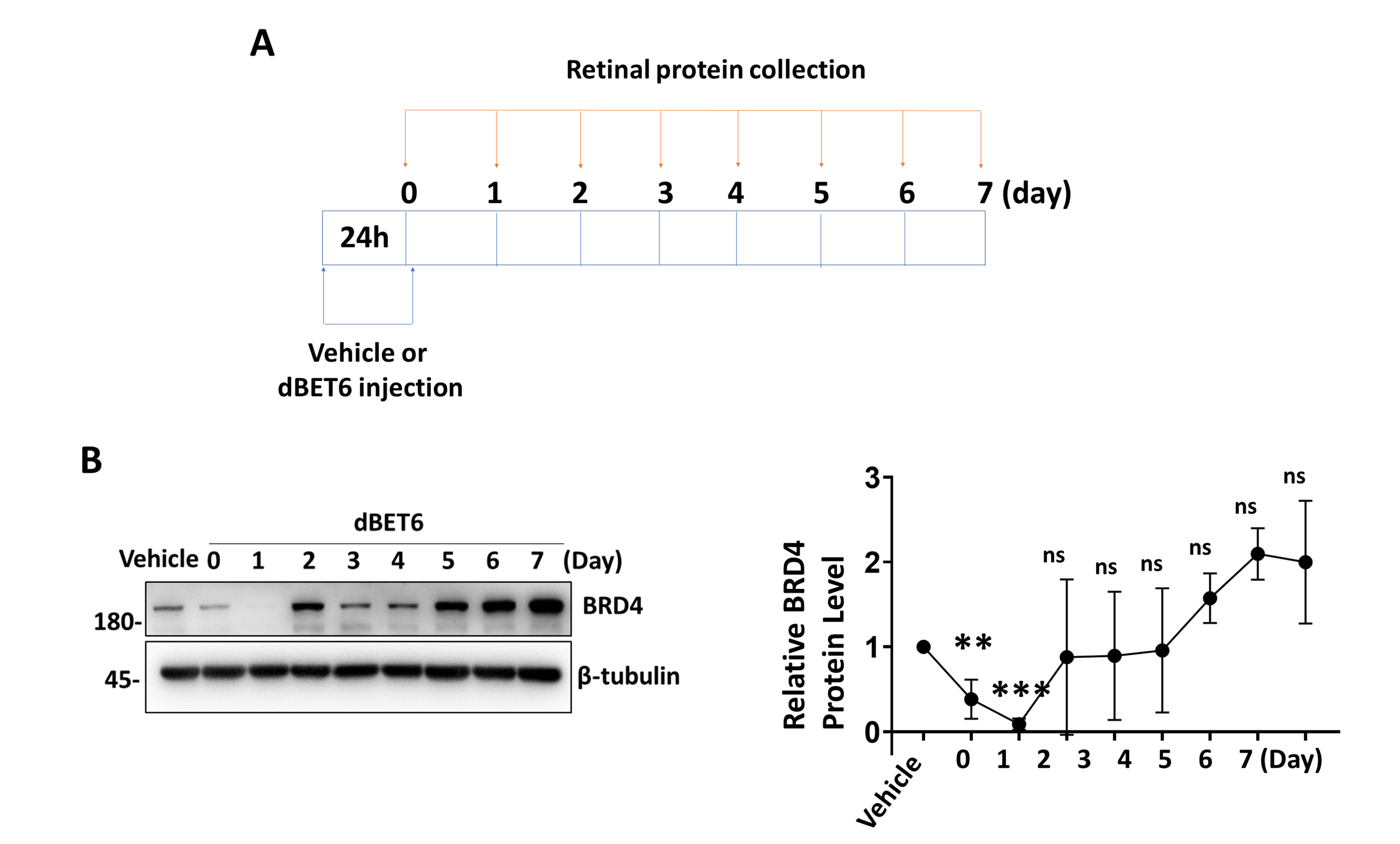

Supplement: Supplementary file 2 — Additional file 2: Figure S2. WB analysis shows retinal BRD4 protein levels. A Schematic diagram shows experimental procedure. For day 0, one i.p. injection of dBET6 was performed and the retinal proteins were collected 24 h post-injection. For other groups, two injections of dBET6 or vehicle were administered and the retinal proteins were extracted at the indicated time points. For vehicle injection, proteins were collected at day 7. B WB analysis of indicated protein. Right panel: quantification of WB. n = 4 eyes per group. **p < 0.01, ***p < 0.0005, One-way ANOVA, Tukey’s test, comparison made between dBET6 samples with Vehicle. [file 12974_2023_2804_MOESM2_ESM.tif]

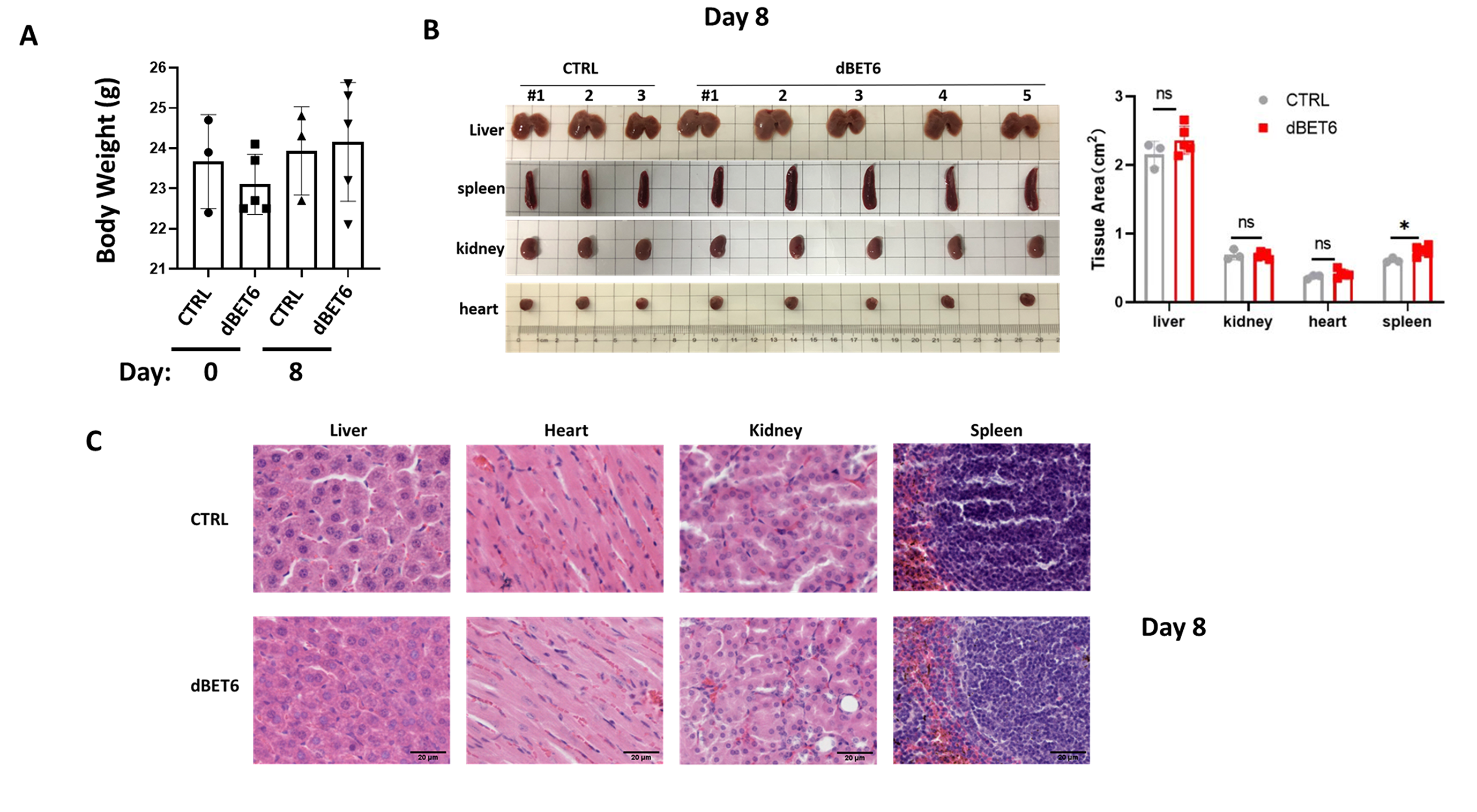

Supplement: Supplementary file 3 — Additional file 3: Figure S3. Photopic ERG recording of vehicle and dBET6-injected mouse retinas. A Representative light-adapted ERG recording. BALB/cJ mice received two injections of vehicle or dBET6 with a 24 h interval. Analysis was conducted 1 dayor 8 daysafter the second injection. B Luminance-response results for the b-waves from mice of indicated treatment. n = 5 mice, 8–10 eyes per group, ns not significant, Two-way ANOVA, Tukey’s test. [file 12974_2023_2804_MOESM3_ESM.tif]

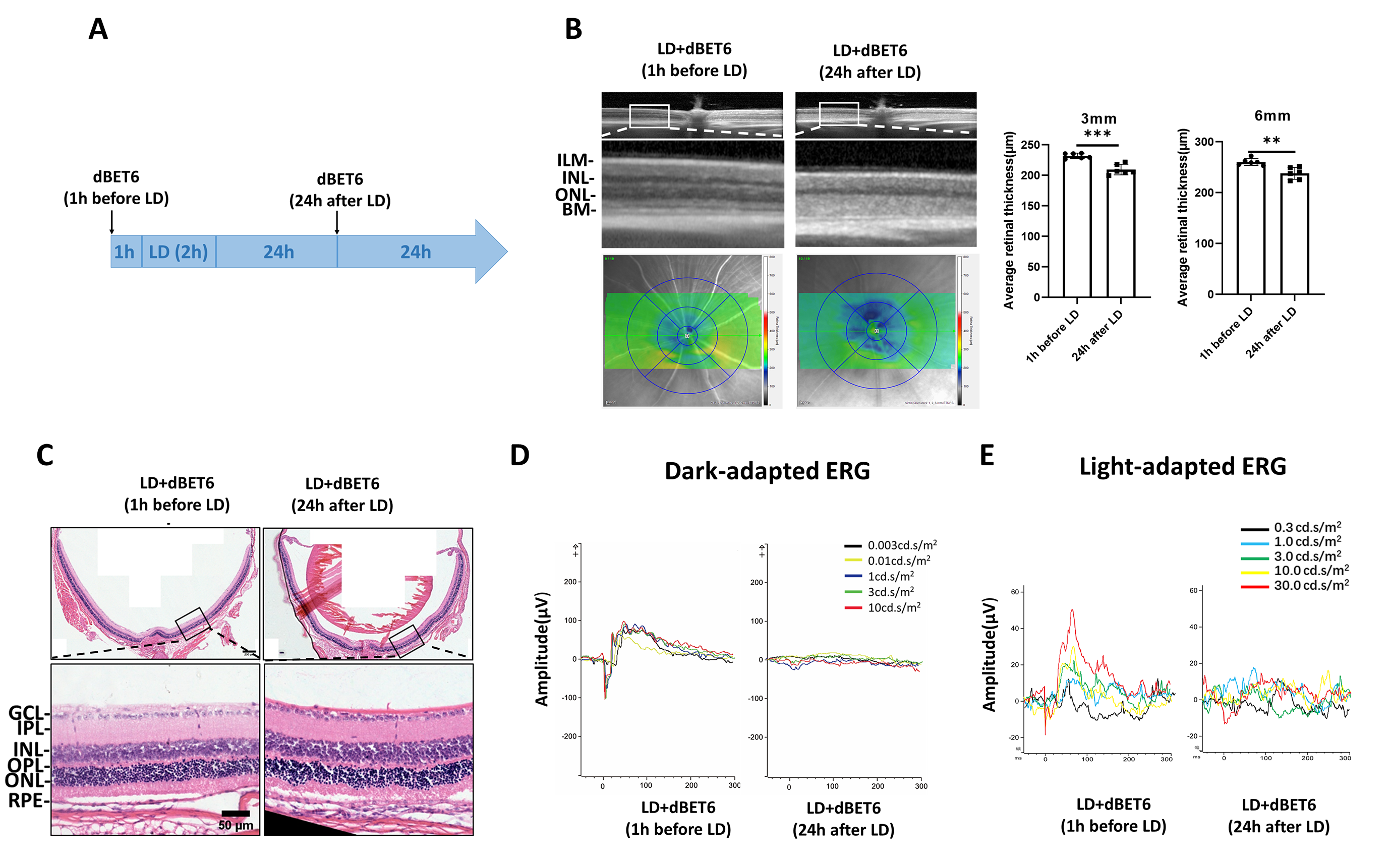

Supplement: Supplementary file 4 — Additional file 4: Figure S4. Systemic administration of dBET6 did not cause obvious organ destruction. I.p. injections of vehicleor dBET6were performed on the 1st and 2nd day of experiment, with 24 h interval. A Mouse body weight beforeand 8 daysafter injections. No significant differences were observed between the vehicle- and dBET6-injected groups. B Left panel: the indicated organs are shown before collection for HE staining 8 days after injection. Right panel: quantification results of the tissue area. The tissue area was calculated by Image J. n = 3 for CTRL and n = 5 for dBET6 treatment. ns not significant, *: p < 0.05, unpaired t-test. C HE staining of the indicated organs. Scale bar: 20 μm. [file 12974_2023_2804_MOESM4_ESM.tif]

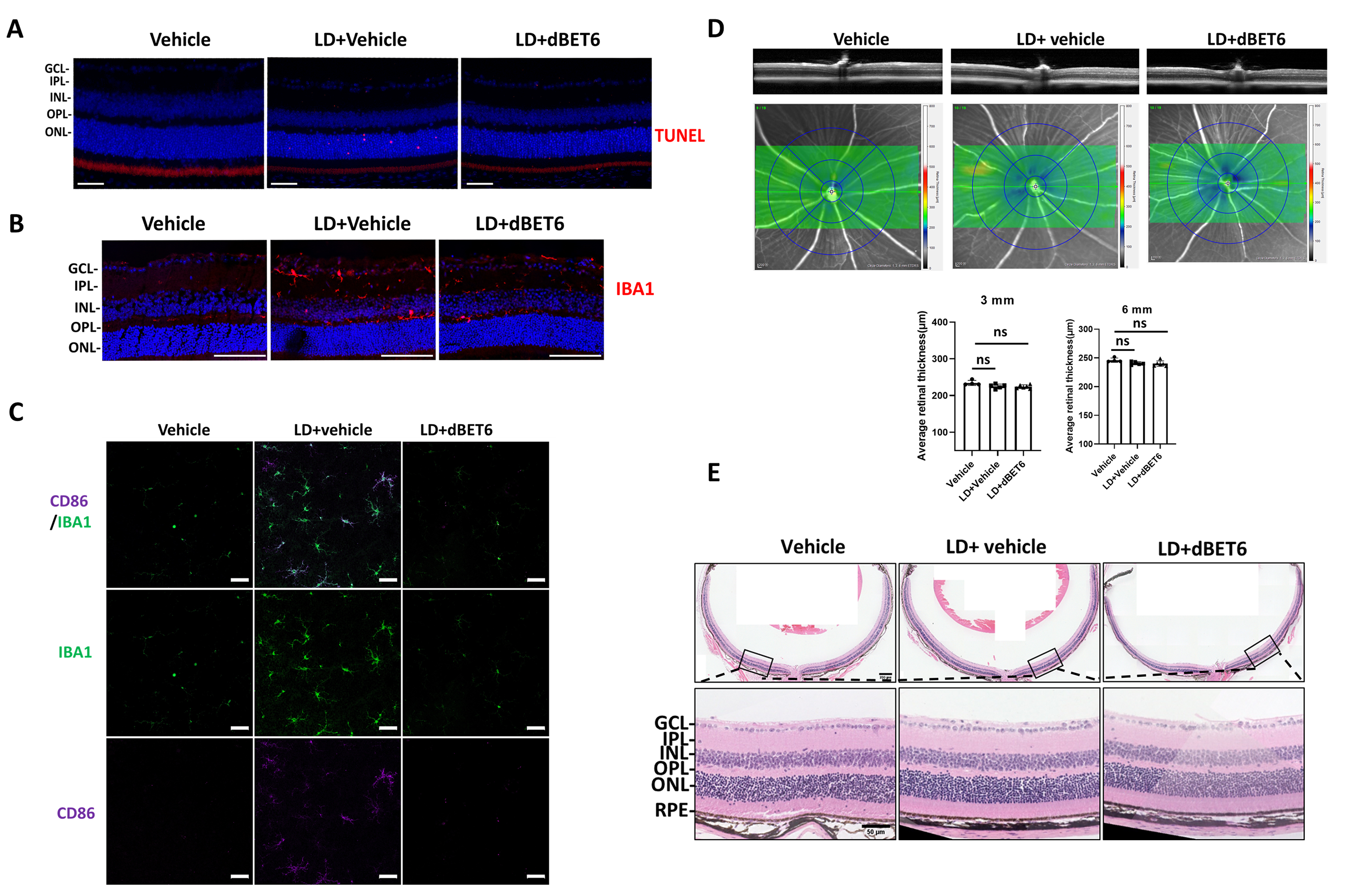

Supplement: Supplementary file 5 — Additional file 5: Figure S5. Photopic ERG recordings of mouse retinas with or without dBET6 treatment. Mice were treated as described in Fig. 2A. A, C Representative light-adapted ERG results in BALB/cJand C57BL/6J. B, D Luminance-response results for the b-waves in BALB/cJand C57BL/6J. ns not significant, #p < 0.05; ## or **p < 0.01; ###p < 0.0005, #### or****: p < 0.0001, two-way ANOVA, Tukey’s test. n = 8–10 mice, 16–20 eyes per group for BALB/cJ; n = 4 mice, 6–8 eyes per group for C57BL/6J. [file 12974_2023_2804_MOESM5_ESM.tif]

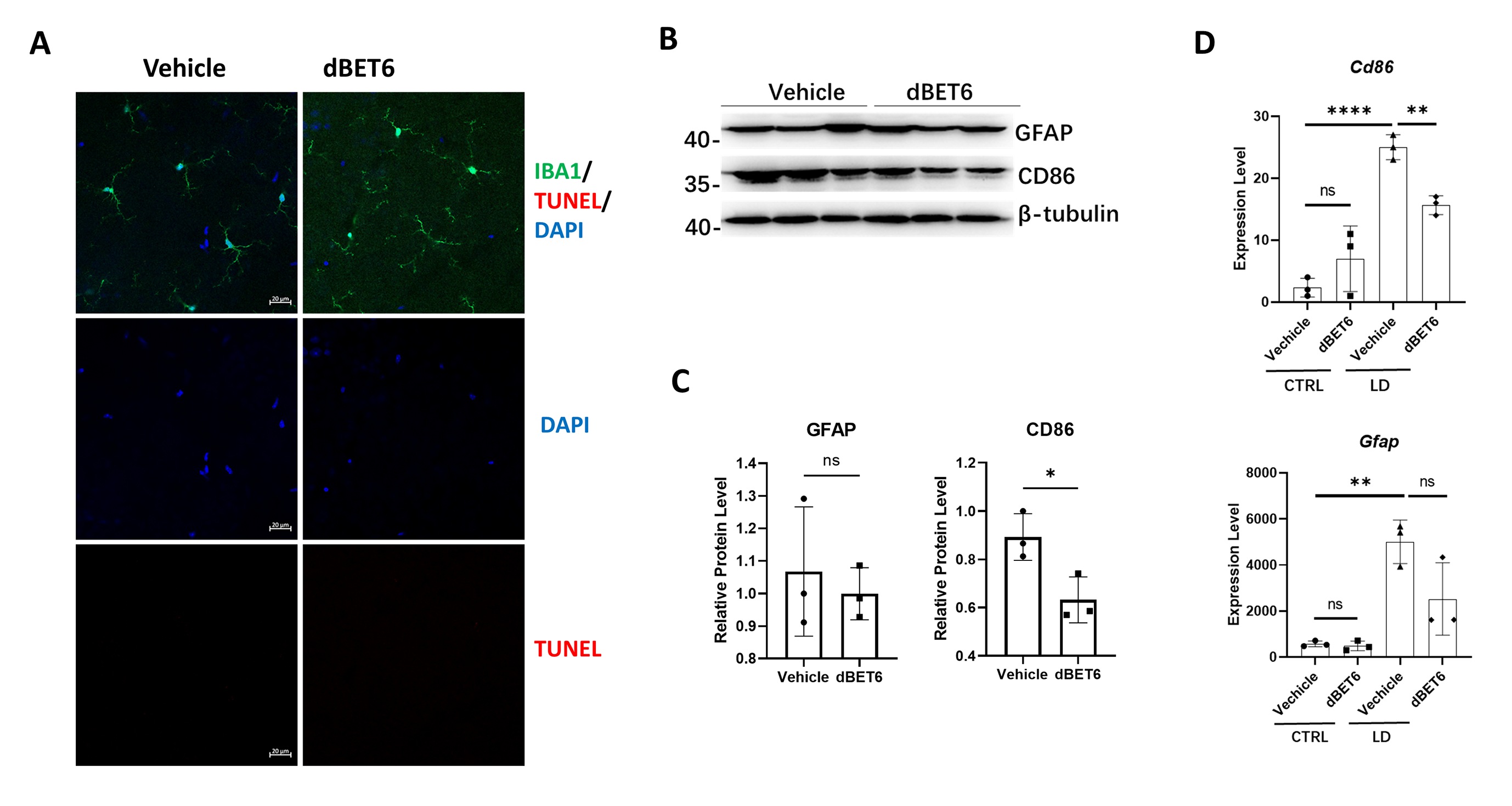

Supplement: Supplementary file 6 — Additional file 6: Figure S6. Retinal morphology and function in mice injected with dBET6 1 h prior to or 24 h after LD. A Schematic diagram shows experimental design. One injection of dBET6was administered at either 1 h before or 24 h after LD. The analysis was performed 48 h post-LD. For LD + vehicle group, vehicle was injected 1 h before LD. B OCT analysis shows in vivo retina morphology. C Quantification of the retinal thickness in 3 mm and 6 mm circles in the OCT images. n = 6 eyes per group, ns not significant, *: p < 0.05, **: p < 0.01, One-way ANOVA, Tukey’s test. D HE staining shows retinal structure. Scale bar: 200 µm for the upper panels and 50 µm for the lower panels. n = 3 eyes per group. E, F. Representative dark-adaptedand light-adaptedERG recording shows retinal response. n = 4 mice, 6–8 eyes per group. [file 12974_2023_2804_MOESM6_ESM.tif]

### Angular Velocities

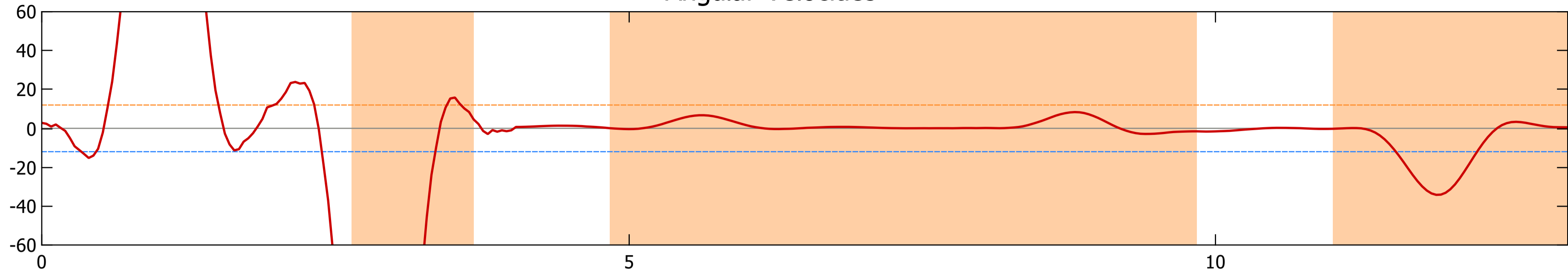

### Tracking Quality

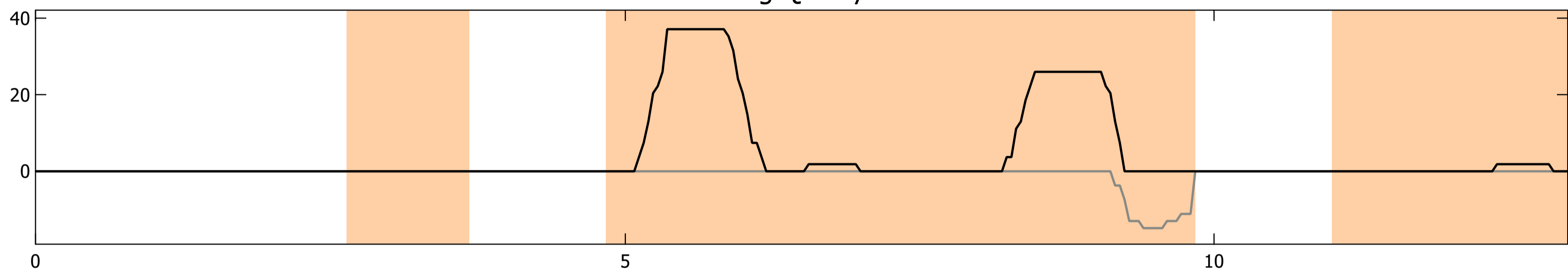

### Score

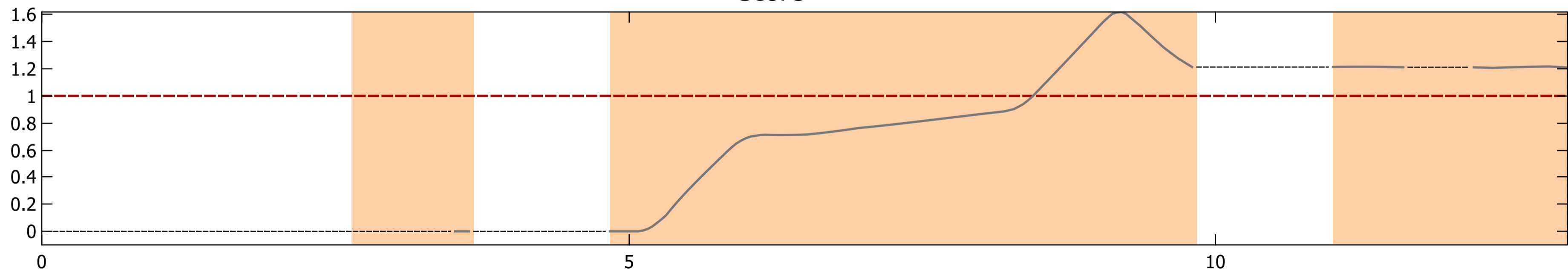

### CW/CCW Contrubition

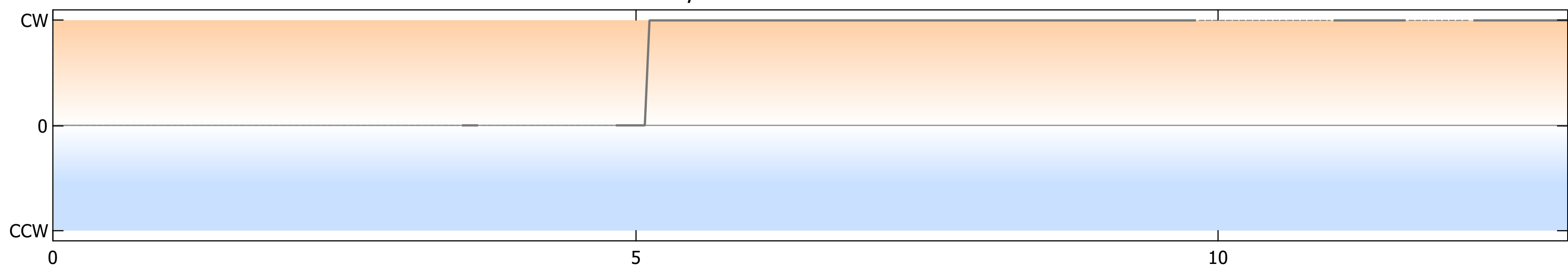

Supplement: Supplementary file 8 — Additional file 8: Figure S8. dBET6 treatment did not cause retinal microglia/macrophages cell death but reduced CD86 protein level. Two injections of vehicle or dBET6 were performed with a 24 h interval. The indicated analysis was conducted at 24 h after the second injection. A IF analysis using retinal flat mounts. The microglia/macrophages were labeled by anti-IBA1 antibody. Note not detectable cell death existed in vehicle or dBET6 treatment, as indicated by TUNEL staining. n = 3 eyes per group, scale bar: 20 μm. B WB analysis of the indicated proteins. C Quantification of WB results. ns not significant, *: p < 0.05, unpaired t-test, n = 3 eyes per group. D RNA-seq result of the indicated genes. The RNA-seq was conducted as described in Fig. 7. ns not significant, **: p < 0.01, ****: p < 0.0001. [file 12974_2023_2804_MOESM8_ESM.zip › 12974_2023_2804_MOESM8_ESM/CTRL-0.472/CTRL-1 (1).pdf]

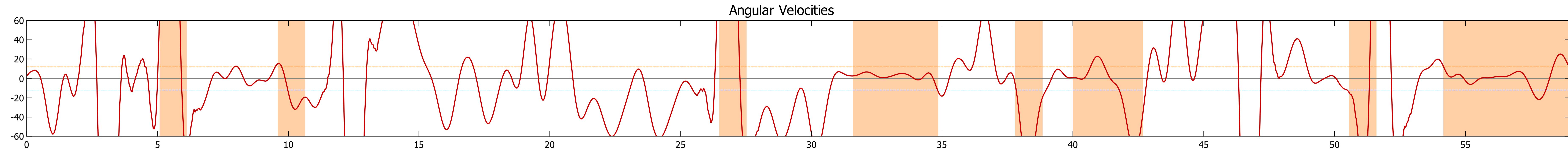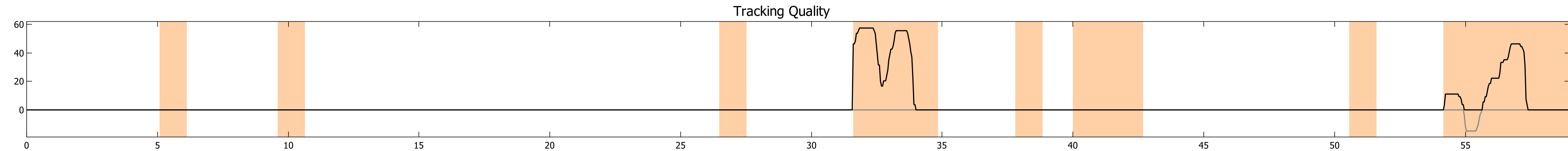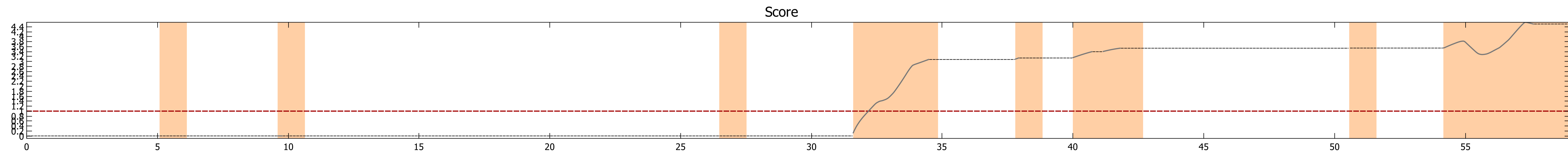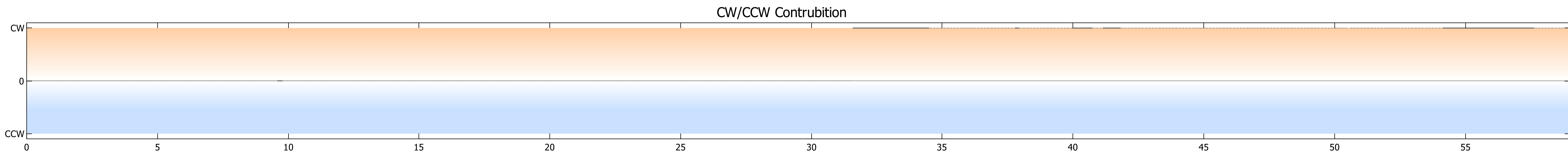

Supplement: Supplementary file 8 — Additional file 8: Figure S8. dBET6 treatment did not cause retinal microglia/macrophages cell death but reduced CD86 protein level. Two injections of vehicle or dBET6 were performed with a 24 h interval. The indicated analysis was conducted at 24 h after the second injection. A IF analysis using retinal flat mounts. The microglia/macrophages were labeled by anti-IBA1 antibody. Note not detectable cell death existed in vehicle or dBET6 treatment, as indicated by TUNEL staining. n = 3 eyes per group, scale bar: 20 μm. B WB analysis of the indicated proteins. C Quantification of WB results. ns not significant, *: p < 0.05, unpaired t-test, n = 3 eyes per group. D RNA-seq result of the indicated genes. The RNA-seq was conducted as described in Fig. 7. ns not significant, **: p < 0.01, ****: p < 0.0001. [file 12974_2023_2804_MOESM8_ESM.zip › 12974_2023_2804_MOESM8_ESM/LD+dBET6/LD+dBET6-1 (1).pdf]

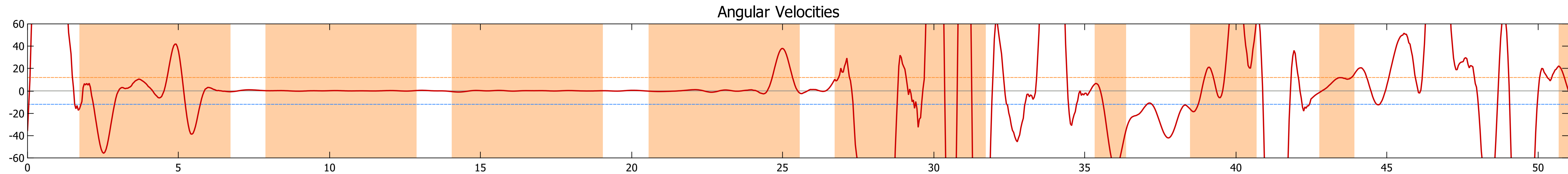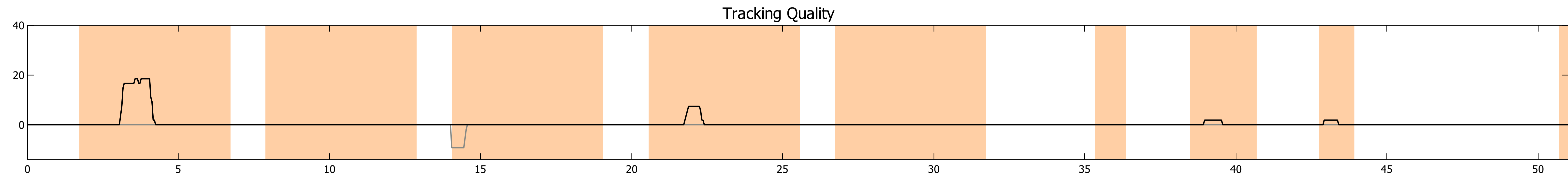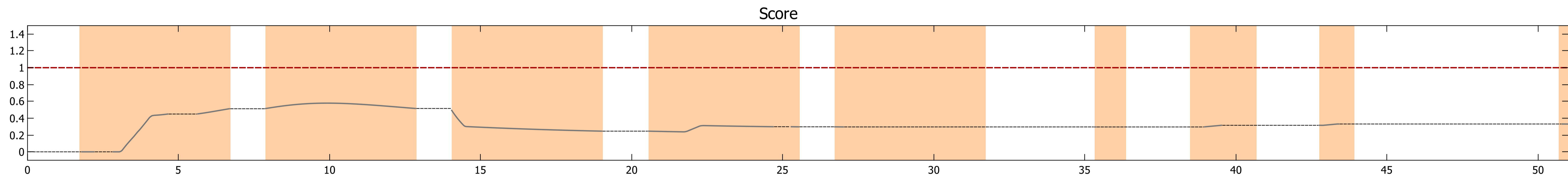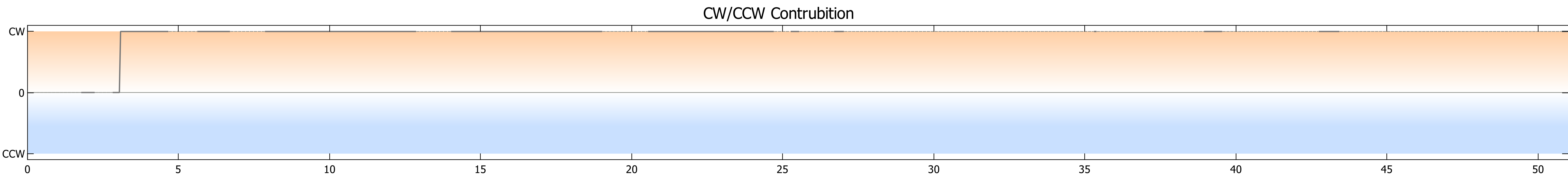

Supplement: Supplementary file 8 — Additional file 8: Figure S8. dBET6 treatment did not cause retinal microglia/macrophages cell death but reduced CD86 protein level. Two injections of vehicle or dBET6 were performed with a 24 h interval. The indicated analysis was conducted at 24 h after the second injection. A IF analysis using retinal flat mounts. The microglia/macrophages were labeled by anti-IBA1 antibody. Note not detectable cell death existed in vehicle or dBET6 treatment, as indicated by TUNEL staining. n = 3 eyes per group, scale bar: 20 μm. B WB analysis of the indicated proteins. C Quantification of WB results. ns not significant, *: p < 0.05, unpaired t-test, n = 3 eyes per group. D RNA-seq result of the indicated genes. The RNA-seq was conducted as described in Fig. 7. ns not significant, **: p < 0.01, ****: p < 0.0001. [file 12974_2023_2804_MOESM8_ESM.zip › 12974_2023_2804_MOESM8_ESM/LD+Vehicle-0.333/LD-1 (1).pdf]

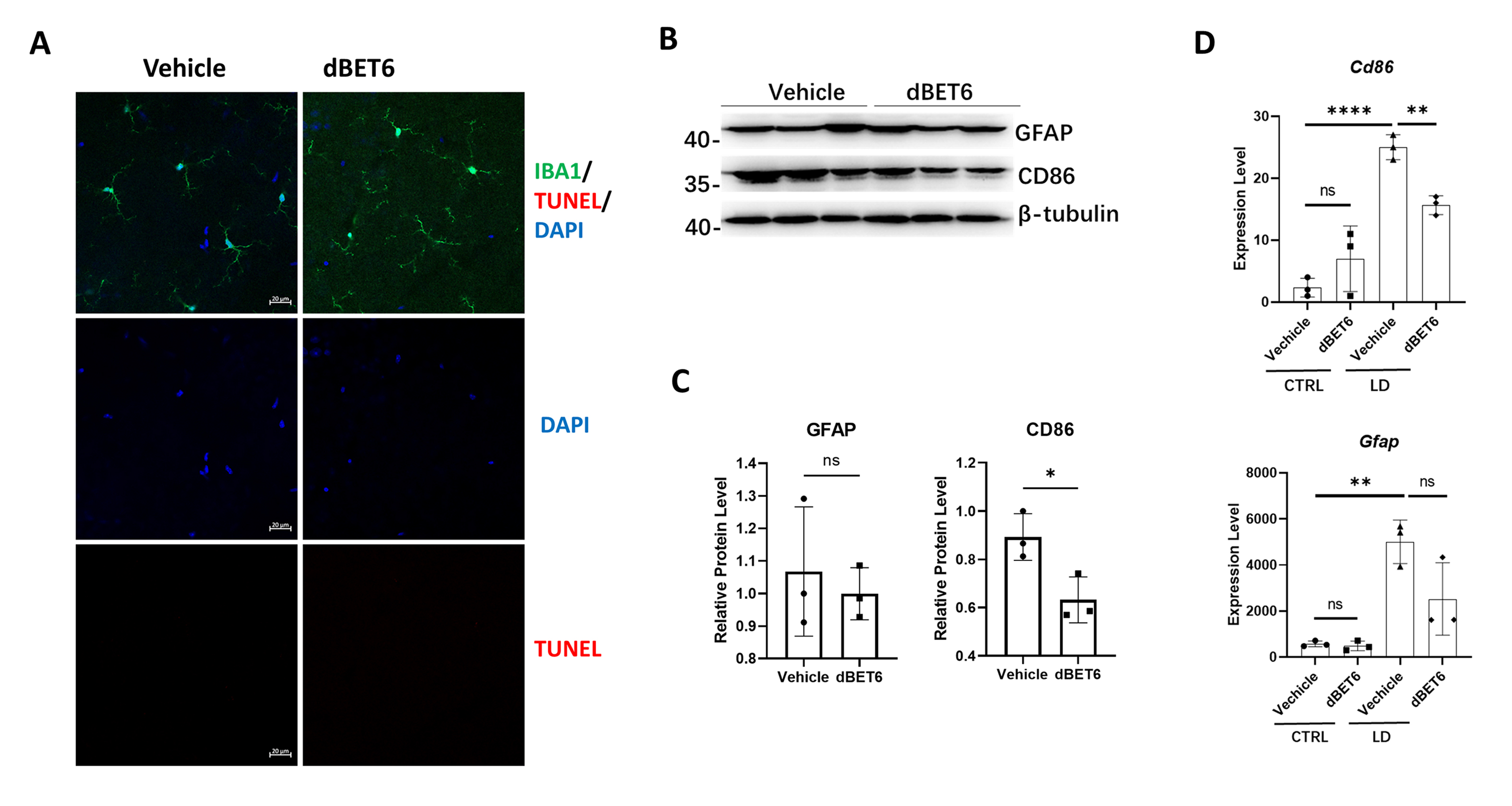

Supplement: Supplementary file 8 — Additional file 8: Figure S8. dBET6 treatment did not cause retinal microglia/macrophages cell death but reduced CD86 protein level. Two injections of vehicle or dBET6 were performed with a 24 h interval. The indicated analysis was conducted at 24 h after the second injection. A IF analysis using retinal flat mounts. The microglia/macrophages were labeled by anti-IBA1 antibody. Note not detectable cell death existed in vehicle or dBET6 treatment, as indicated by TUNEL staining. n = 3 eyes per group, scale bar: 20 μm. B WB analysis of the indicated proteins. C Quantification of WB results. ns not significant, *: p < 0.05, unpaired t-test, n = 3 eyes per group. D RNA-seq result of the indicated genes. The RNA-seq was conducted as described in Fig. 7. ns not significant, **: p < 0.01, ****: p < 0.0001. [file 12974_2023_2804_MOESM8_ESM.zip › 12974_2023_2804_MOESM8_ESM/R2-Fig. S8 CD86 and GFAP in ctrl and bet6 retina.jpg]

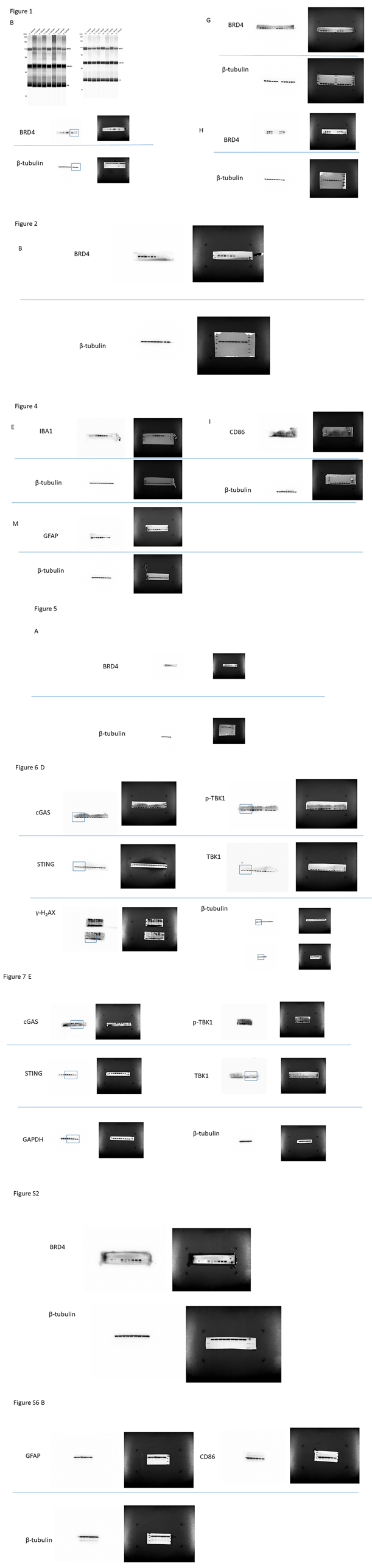

Supplement: Supplementary file 9 — Additional file 9: Figure S9. Original WB images in this study. The uncropped WB, and the images merged with the protein marker are shown. [file 12974_2023_2804_MOESM9_ESM.jpg]
